# Statistical Inference of In Vivo Properties of Human DNA Methyltransferases from Double-Stranded Methylation Patterns

**DOI:** 10.1371/journal.pone.0032225

**Published:** 2012-03-19

**Authors:** Audrey Q. Fu, Diane P. Genereux, Reinhard Stöger, Alice F. Burden, Charles D. Laird, Matthew Stephens

**Affiliations:** 1 Department of Physiology, Development and Neuroscience, Cambridge Systems Biology Centre, University of Cambridge, Cambridge, United Kingdom; 2 Department of Biology, University of Washington, Seattle, Washington, United States of America; 3 School of Biosciences, University of Nottingham, Sutton Bonington Campus, Leicestershire, United Kingdom; 4 Departments of Human Genetics and Statistics, University of Chicago, Chicago, Illinois, United States of America; Université Paris-Diderot, France

## Abstract

DNA methyltransferases establish methylation patterns in cells and transmit these patterns over cell generations, thereby influencing each cell's epigenetic states. Three primary DNA methyltransferases have been identified in mammals: DNMT1, DNMT3A and DNMT3B. Extensive in vitro studies have investigated key properties of these enzymes, namely their substrate specificity and processivity. Here we study these properties in vivo, by applying novel statistical analysis methods to double-stranded DNA methylation patterns collected using hairpin-bisulfite PCR. Our analysis fits a novel Hidden Markov Model (HMM) to the observed data, allowing for potential bisulfite conversion errors, and yields statistical estimates of parameters that quantify enzyme processivity and substrate specificity. We apply this model to methylation patterns established in vivo at three loci in humans: two densely methylated inactive X (Xi)-linked loci (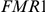 and 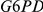), and an autosomal locus (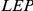), where methylation densities are tissue-specific but moderate. We find strong evidence for a high level of processivity of DNMT1 at 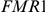 and 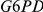, with the mean association tract length being a few hundred base pairs. Regardless of tissue types, methylation patterns at 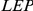 are dominated by DNMT1 maintenance events, similar to the two Xi-linked loci, but are insufficiently informative regarding processivity to draw any conclusions about processivity at that locus. At all three loci we find that DNMT1 shows a strong preference for adding methyl groups to hemi-methylated CpG sites over unmethylated sites. The data at all three loci also suggest low (possibly 0) association of the de novo methyltransferases, the DNMT3s, and are consequently uninformative about processivity or preference of these enzymes. We also extend our HMM to reanalyze published data on mouse DNMT1 activities in vitro. The results suggest shorter association tracts (and hence weaker processivity), and much longer non-association tracts than human DNMT1 in vivo.

## Introduction

DNA methyltransferases establish methylation patterns in cells and transmit these patterns over cell generations, thereby influencing each cell's epigenetic states. (See [1] for an overview of methyltransferases, and Supplementary Material of [2] for an introduction to DNA methylation aimed at non-biologists.) Three primary DNA methyltransferases have been identified in mammals: DNMT1, DNMT3A and DNMT3B [3],[4]. Whereas the DNMT3s are mostly responsible for establishing methylation patterns during early development and are therefore commonly known as the de novo methyltransferases, DNMT1 is mostly responsible for maintaining existing methylation patterns over somatic cell divisions, and is therefore commonly known as the maintenance methyltransferase [1].

**Figure 1:**
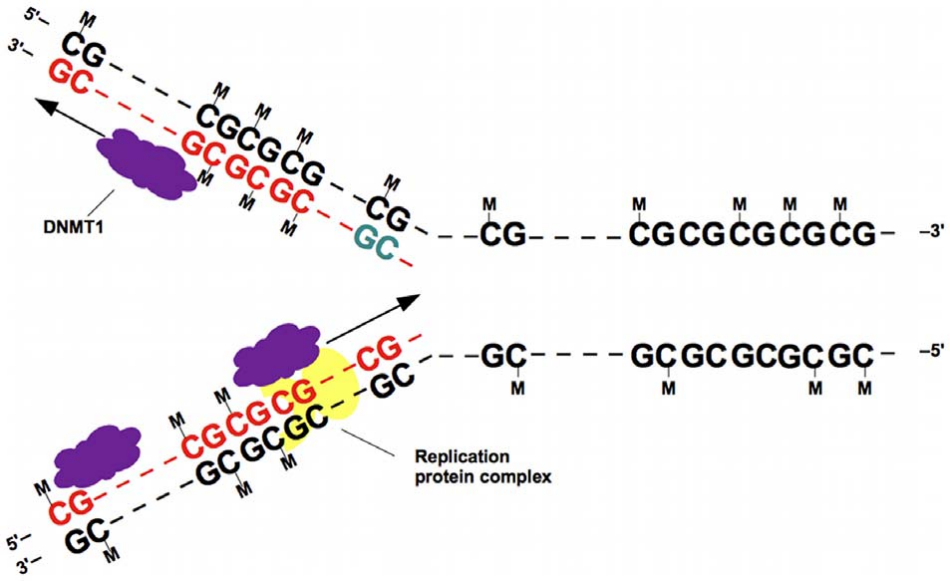
Model for processivity of methyltransferase DNMT1 following DNA replication. Newly synthesized daughter strands (red, mostly, and pale green) are initially unmethylated. Following DNA replication, DNMT1 (purple) binds to the hemimethylated CpG dyads, perhaps with the aid of the replication protein complex (yellow), which includes proteins PCNA and UHRF1 [1]. DNMT1 is proposed to move processively along the molecule from the 5′ end to the 3′ end of the daughter strand. Note that the leftmost CpG dyad at the top is hemimethylated, because DNMT1 has not reached it yet. Methylation by DNMT1 is not perfect (e.g., the CpG in pale green remains unmethylated), because DNMT1 either fails to add methyl groups (denoted by letter M) to its associated CpG dyads, or is unassociated with the DNA at those sites.

A central component of the widely accepted model for the maintenance of DNA methylation in eukaryotes is processive actions of the maintenance methyltransferase DNMT1 at hemimethylated CpG dyads shortly after DNA replication (Figure 1; [1]). This model relies on two properties of DNMT1: substrate specificity (i.e., acting in different ways or at different rates on different types of substrate) and processivity (i.e., associating consecutively at multiple sites along the DNA). These are key properties of DNA methyltransferases and many other DNA-binding enzymes [1], [5], and both properties have been investigated extensively in vitro.

Regarding substrate specificity, in vitro experiments show that DNMT1 preferentially adds methyl groups to the cytosines in daughter-strand CpGs that pair with methylated, rather than unmethylated, parent-strand CpGs (i.e., hemimethylated CpG dyads), thus maintaining methylation at these CpG sites over cell generations [6]. Such a preference for hemimethylated CpG dyads was predicted for maintenance methyltranferases as early as 1968 [7], and is now commonly measured in terms of the “hemi-preference ratio”. This ratio represents the relative rates with which an enzyme methylates hemimethylated and unmethylated CpG dyads. Reported estimates for DNMT1 in humans and mice, generally from in vitro experiments, vary widely from 2- to 200-fold, depending on the DNA sequence context, experimental conditions and enzyme preparation [6].

Regarding processivity, in vitro experiments suggest that mouse DNMT1 acts processively, binding to DNA and then remaining active over a stretch of consecutive nucleotides [1], [6]. Both human and mouse orthologs of DNMT1 have been found to associate with the DNA replication machinery, which includes proteins PCNA and UHRF1 [8], [9]. The DNMT1s are thus poised to methylate cytosines shortly after their incorporation into the nascent daughter DNA strand. However, experiments indicate that both mouse and human orthologs can also processively modify hemimethylated dyads in synthetic DNAs in the absence of the replication machinery [10]–[14]. This result suggests that the interaction of the human and mouse orthologs with the replication machinery may not be essential to enzyme activities.

De novo methyltranferases DNMT3A and 3B [4] are also important in the preservation of appropriate epigenetic states in human and mouse somatic cells [15]. The absence of these methyltransferases can lead to abnormal phenotypes [16], [17]. In vitro experiments have also investigated substrate specificity and processivity of these enzymes. Regarding substrate specificity, in contrast to DNMT1, neither DNMT3A nor 3B show preference for adding methyl groups to hemimethylated CpG dyads over unmethylated dyads [4], [18]. Studies of possible processivity of the DNMT3s are less extensive than for DNMT1. In vitro experiments have demonstrated non-processive behavior of mouse DNMT3A but highly processive behavior of mouse DNMT3B [19], and processive behavior of human DNMT3A [20].

Despite the availability of significant in vitro data, important questions remain to be addressed regarding the in vivo properties of the DNA methyltransferases. Here, we investigate in vivo substrate preferences and levels of processivity of human DNMT1 and DNMT3s by analyzing double-stranded DNA methylation patterns established in vivo, measured using hairpin-bisulfite PCR [21], [22]. Previous analyses of some of these double-stranded patterns [2], [23] yielded estimates of CpG site-specific rates of maintenance methylation and parent- and daughter-strand de novo methylation. However, these previous analyses aimed at quantifying the outcome of the methylation process, without consideration of the enzymes invovled. Here, we develop a novel Hidden Markov Model (HMM) to account for the properties of the DNMTs responsible for the methylation process. Our HMM includes parameters that capture both substrate specificity and processivity of each enzyme, and hence allows inference of these parameters from observed data. In addition to these in vivo analyses, we also apply the HMM (suitably modified) to re-analyze several published in vitro data sets, and compare with our in vivo results.

**Figure 2:**
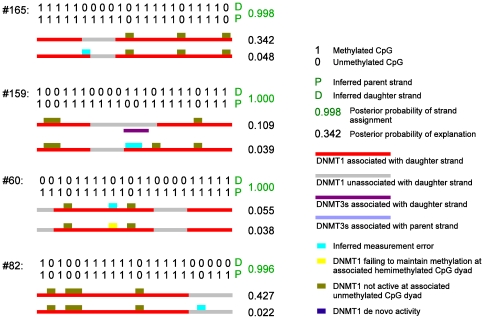
Four most informative *FMR1* methylation patterns and inference for each of them under HMM. Each pattern contains a pair of parent and daughter strands; which is the parent strand and which is the daughter strand is not known directly. Our HMM infers probabilistically the strand assignments for each pattern. These posterior probabilities are shown in green for the indicated parent and daughter strand assignment (indicated by letters P and D in green). These patterns from our *FMR1* data contain long runs of hemimethylated CpG dyads with their methyl groups present on the same strand. The top two most likely explanations, among all 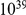 possible explanations, are shown for each pattern. Symbols indicate possible states and activities of the methyltransferases. Not all symbols are used here. The effect of these four patterns on parameter estimation is further shown in Supplementary Figure 13 in Materials S1.

An important feature of our analyses is that they are based on a statistical model, which quantifies processivity as a probability, thus allowing for statistically testing whether processivity exisits, and which assesses the statistical uncertainty in the estimate of this probability, thus facilitating comparison of inferred levels of processivity among data sets. Our HMM also explicitly accounts for potential measurement errors in the observed data; these errors have been generally unaccounted for in other in vivo and in vitro studies. Additionally, this HMM can also be used to infer the set of enzymatic activities that most likely gave rise to each observed methylation pattern, and to infer, probabilistically, which strand in each double-stranded methylation pattern is the parent strand, and which is the daughter strand (this information is not directly measurable in the hairpin-bisulfite experiment), allowing for investigation of strand-specific behavior of these enzymes (Figure 2).

## Results

### A hidden Markov model (HMM) for processivity and other properties of the DNMTs

We model the observed double-stranded methylation patterns as having arisen from a process where the methyltransferases (DNMT1 and the DNMT3s) were either associated or not associated with the DNA at a CpG site. We model these association/non-association states as a Markov process along the DNA. The model for the observed data based on these unobserved (“hidden”) states is then a Hidden Markov Model (HMM [24]). For in vivo data, we cannot rule out that other (perhaps unidentified) enzymes than DNMT1 and the DNMT3s may have also contributed to the observed methylation patterns. To allow for this, our references to DNMT1 could be interpreted broadly as referring to the enymes whose activities are primarily maintenance methylation, and references to DNMT3s could be interpreted broadly as enzymes whose activities are primarily de novo methylation.

More specifically, the hidden Markov process in the HMM can be decomposed into three independent Markov processes: the first representing association of DNMT1, which we assume to act only on the daughter strand [10], the second representing the association of the DNMT3s on the parent strand, and the third representing the association of the DNMT3s on the daughter strand. We use the subscripts 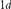, 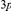 and 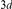 to refer to each of these processes. We characterize each Markov process by two transition probabilities: the reassociating probability per bp, 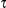, that unassociated molecules of the methyltransferase become associated with DNA over 1 bp, and the dissociating probability per bp, 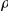, that associated molecules of the methyltransferase become unassociated from DNA over 1 bp. The transition probability matrix over 1 bp for each Markov process can then be written as in Eq. (1).
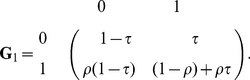
(1)The transition probability matrix between two CpG sites 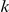 bp apart is then 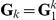.

Under this parameterisation, two events contribute to the probability of a methyltransferase staying associated at two consecutive sites 1 bp apart (i.e., the bottom right entry in 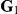): (i) processivity, whereby the methyltransferase stays associated from site 1 to site 2 without ever dissociating from DNA. This happens with probability 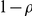; and (ii) dissociation-reassociation events, whereby the methyltransferase is associated at site 1, then “falls off”, but becomes re-associated with DNA at site 2. This happens with probability 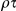. We quantify the strength of processivity, which corresponds to event (i), by the dissociating probability 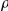: the value 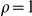 corresponds to “no processivity”, where the methyltransferase dissociates from DNA at every base pair and the association state is independent at each site (i.e., the two rows of 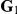 are identical); values of 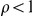 correspond to processive behaviour, and the smaller the value of 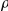, the stronger the processivity. This parameter translates into the expected length of each association tract of 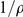 bp, which is a more conventional measure of processivity and quantifies processivity directly in terms of tract length. Because 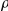 concerns only enzyme molecules that are already associated, this mean length of association tracts excludes multiple dissociation-reassociation events at consecutive CpG sites, which could be mistaken for processivity. Similarly, the expected length of non-association tracts, which are gaps between association tracts, has mean 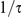 bp. Unlike 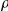, the reassociating probability 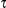 could be driven principally by concentrations of unassociated methyltransferase molecules in the nucleus.

Parameters 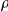 and 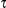 together determine the average frequency with which the methyltransferase is associated with the DNA at each CpG site. Using 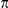 to denote this frequency, we have
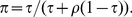
(2)


In addition to these parameters relating to processivity, our model also has parameters for the methylation activities of the methyltransferases when they are associated with DNA, and parameters for the measurement errors that can result from bisulfite conversion (see Materials and Methods for detail). Together these parameters, the “emission probabilities” of the HMM, describe how association or non-association states of methyltransferases give rise to observed methylation states on the pairs of parent and daughter strands, subject to measurement errors (see Materials and Methods for detail). We allow the methyltransferases to methylate daughter CpGs at associated hemi-methylated sites with probability 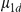 for DNMT1 (or 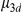 for the DNMT3s) and at associated unmethylated sites with probability 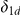 for DNMT1 (or 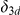 for the DNMT3s). The ratios 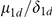 and 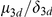 are termed the “hemi-preference ratios” for DNMT1 and the DNMT3s, respectively. For the parent strand, we make the simpler assumption that the DNMT3s always add a methyl group to the associated CpG.

The above model is very general, allowing for a complex combination of behaviors of the methyltransferases. In applications, it can be helpful to constrain this model in various ways, either to deal with data collected from particular experimental conditions, or to make parameters more identifiable. For example, imposing the constraint 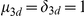 yields a more parsimonious model in which the DNMT3s always methylate the associated daughter-strand CpG. This is the model we use for most analyses presented here. However, to attempt to estimate the hemi-preference ratio for the DNMT3s, we impose a different constraint 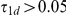 and 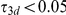 onto the general model. This constraint reflects the setting where DNMT1 is the primary maintenance enzyme, and helps to distinguish the DNMT1 process and the daughter-strand process of the DNMT3s, which would have been indistinguishable otherwise (see Materials and Methods for detail). To analyze the in vitro data on DNMT1 under the same model, we estimate parameters only associated with the DNMT1 process, and fix the reassociating probabilities 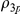 and 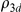 to be 0 and the dissociating probabilities 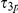 and 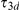 to be 1; these constraints reflect the in vitro setting where the DNMT3s are absent.

We fit the HMM to the data in a Bayesian inference framework [25], using Markov chain Monte Carlo (MCMC) to produce samples from the joint posterior distribution of all parameters in the model given the data (see Materials S1 for details, including specification of relevant prior distributions). At the core of our implementation is the standard forward-backword algorithm in each MCMC iteration for computing the joint likelihood of the parameters given observed methylation patterns. The computational complexity of the forward-backward algorithm for all 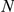 patterns across 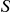 CpG sites in each MCMC iteration is 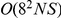, where 8 (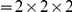) is the number of hidden states at each site, with 2 being the two states (associated and unassociated) of each Markov process. We summarize the posterior distributions of the parameters from the Bayesian inference by the posterior median and 80% credible intervals (80% CIs; 10- and 90-percentiles); 80% intervals were used, rather than more conventional 95% intervals, to reduce the impact of the heavy tails of some distributions. This inference procedure accounts for the uncertainty in the data regarding which enzymatic activities produced each observed double-stranded pattern by using a dynamic programming algorithm to sum over all possibilities, weighting each possibility by its probability (Figure 2; see also Materials S1).

### Runs of hemi-methylated dyads provide information on processivity

As mentioned above, both processivity and multiple dissociation-reassociation events may give rise to runs of fully methylated dyads. When the observed patterns contain only runs of fully or un-methylated dyads, we cannot tell these two mechanisms apart. Presence of hemimethylated dyads (“hemis” hereinafter) provides additional information. Whereas randomly-distributed hemis in the data suggest that the methyltransferases may have been associated with or dissociated from the dyads randomly, clustered hemis suggest nonrandom enzyme activities. In particular, runs of hemis of the same orientation (i.e., methylated CpGs appearing on the same strand), if observed more often than expected by chance, provide evidence for processivity. For example, the in vitro data on mouse DNMT1 from Goyal et al. [11] (their Fig. 3A) shows multiple very long runs of hemi-methylated sites of the same orientation. Some of these runs contain as many as 20 hemimethylated dyads, with the parent strands being methylated prior to the reaction with DNMT1. These long runs are extremely unlikely if DNMT1 were to associate with the DNA independently at each site. In our in vivo data, similar runs of hemis, although much shorter, also suggest the presence of processivity: indeed a permutation test based on correlations at adjacent CpG sites produces a 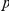 value of 0.002, suggesting that these runs of hemis occur more often than expected by chance; see Supplementary Figure 1 in Materials S1. Observations like this motivated the HMM described above, and results from the HMM confirm that these runs of hemi-methylated sites are likely due to DNMT1 being unassociated with the DNA for several sites in succession (see Figure 2 for the top two explanations our HMM inferred for four patterns collected at the 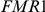 locus).

**Figure 3:**
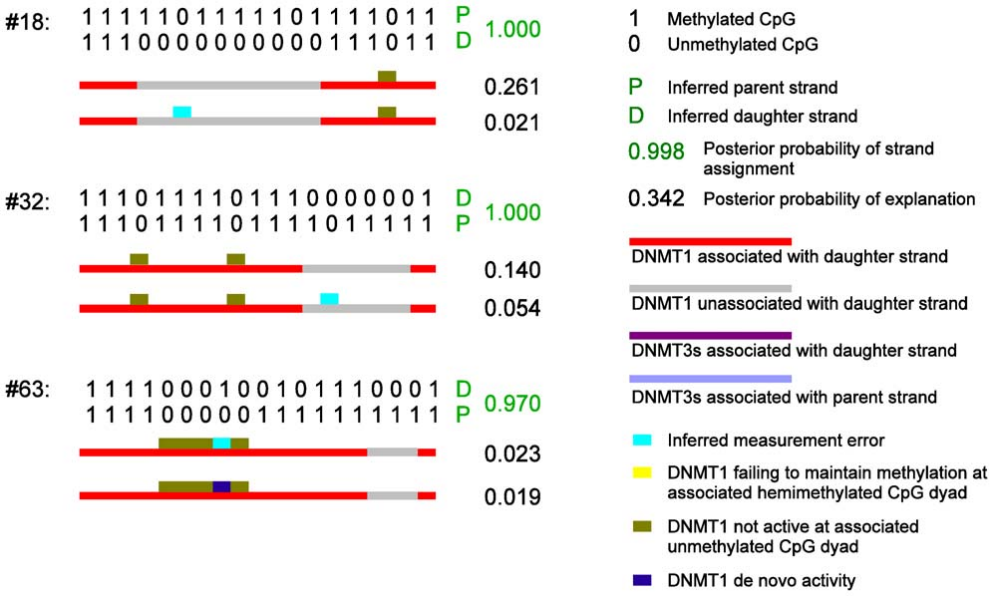
Three most informative *G6PD* methylation patterns and inference for each of them under HMM. Symbols are as in Figure 2.

### Strong processivity and high hemi-preference ratio of in vivo human DNMT1 at two inactive X (Xi)-linked loci

The methylation patterns at the 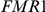 and 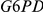 loci share many similarities: 77–82% of the CpG sites are fully methylated, whereas only 6–9% are hemimethylated, some of which form a few relatively long runs of hemimethylated dyads (Table 1). Analyses of these two data sets under our HMM also produce similar estimates of the key parameters.

**Table 1:** Features of the three loci and summary statistics of the methylation patterns in the four human in vivo data sets.

	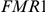	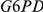	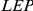	
			In Fat	In Blood
Genomic location	ChrX: 146,800,867-1,008	ChrX: 153,775,537-698	Chr7: 127,881,204-375	
Region length (bp)	142	122	172	
No. of CpG sites	22	19	21	
Median distance (bp)	6.7	6.5	7.5	
between CpG sites				
No. of ds patterns	169	75	80	34
% of M, H, U*	(82, 6, 12)	(77, 9, 14)	(16, 4, 80)	(40,5,55)
Runs of 2 hemis**	11	4	6	1
Runs of 3 hemis	1	1	1	0
Runs of 4 hemis	3	1	0	0
Runs of 5 hemis	0	1	0	0
Runs of 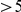 hemis	0	0	0	0

* Percentages of methylated, hemimethylated and unmethylated dyads.

** Consecutive hemimethylated CpG dyads with methylated groups appearing on the same strand.

**Figure 4:**
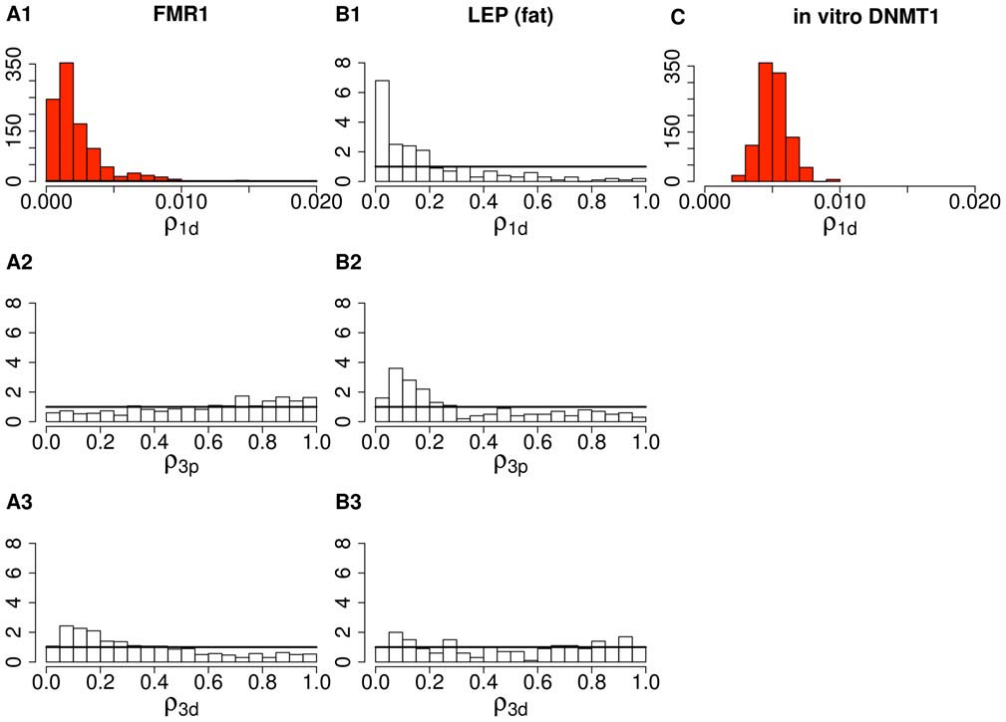
Posterior distributions of dissociating probability 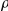 from in vivo methylation data at several loci. From top to bottom, each row indicates 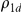 for DNMT1 on the daughter strand, 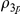 for the DNMT3s on the parent strand, and 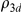 for the DNMT3s on the daughter strand. (**A1**)–(**A3**) are estimated for the 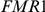 locus. Estimates for 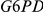, the other Xi-linked locus, show similar distributions and are not displayed here. (**B1**)–(**B3**) are estimated for the autosomal 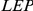 locus in the fat tissue. Estimates for the same locus in the blood tissue show similar distributions and are not displayed here. (**C**) is estimated from the data in Goyal et al. [11], one of the four in vitro data sets on mouse DNMT1 we analyze here. Dark lines indicate the density of a uniform (0,1) prior distribution. Except for the plots in red, which have most probability mass around small values, all the other plots are displayed on the same support of (0,1).

We find that the methylation patterns at both of these Xi-linked loci provide strong evidence for substantial processivity of DNMT1: the estimated dissociating probability for the DNMT1 process, 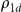, is concentrated on small values near 0 (Table 2; Figure 4(A1)). To investigate the robustness of the estimates, we further ran our HMM on the 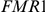 data with different prior distributions assigned to 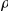. The estimates were similar across these runs (Supplementary Figure 2 in Materials S1). These estimates of 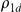 at these two loci imply a mean association tract length of around 600 bp, which is equivalent to about 90 CpG sites (note that there is considerable uncertainty in these estimates, and the 80% CIs span almost 200–2000 bp; Table 2). This inferred length is much greater than the genomic regions covered by our data (142 bp at 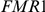 and 122 bp at 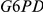; Table 1), as it reflects the expect length of DNMT1 association, had we measured methylation over a much longer genomic region (Figures 2 and 3). Interpreting estimated processivity in terms of tract length allows us to compare our estimates directly with other estimates reported in the literature. On the other hand, we estimate at both loci with strong evidence that the reassociating probability 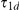 is not high, with the median being 0.12 at 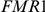 and 0.07 at 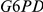 and the 80% CIs being narrow (Table 2; also see Supplementary Figures 3 and 4 in Materials S1). This estimate is also robust to different priors (Supplementary Figure 5 in Materials S1). The high association tract length and low reassociating probability for these two hypermethylated loci imply that strong processivity, rather than random association, of DNMT1 accounts for most of the runs of fully methylated CpG dyads.

**Table 2:** Parameter estimates under our HMM for the four human in vivo data sets.

			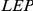 (Chr 7)	
Parameter	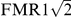 (Chr X)	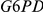 (Chr X)	In Fat	In Blood
Mean association				
length (bp):				
of DNMT1	597 (219–1521)	673 (238–1874)	9 (2–88) 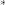	9 (2–89) 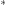
of DNMT3s on parent	2 (1–6) 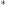	1 (1–3) 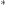	5 (1–17) 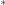	1 (1–4) 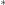
of DNMT3s on daughter	4 (1–14) 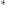	1 (1–3) 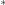	2 (1–14) 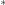	2 (1–3) 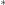
Dissociating				
probability:				
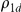	0.002 (0.000–0.005)	0.001 (0.001–0.004)	0.11 (0.01–0.53) 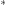	0.11 (0.01–0.63) 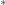
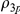	0.65 (0.16–0.93) 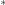	0.71 (0.35–0.94) 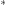	0.20 (0.06–0.82) 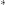	0.68 (0.28–0.92) 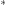
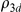	0.28 (0.07–0.79) 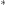	0.72 (0.38–0.95) 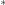	0.49 (0.07–0.93) 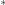	0.66 (0.29–0.92) 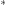
Reassociating				
probability:				
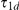	0.12 (0.07–0.21)	0.07 (0.03–0.12)	0.81 (0.21–0.96) 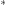	0.91 (0.60–0.98) 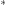
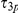	0.01 (0.00–0.03)	0.03 (0.00–0.08)	0.00 (0.00–0.00)	0.00 (0.00–0.01)
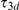	0.01 (0.00–0.03)	0.03 (0.00–0.10)	0.00 (0.00–0.00)	0.01 (0.00–0.02)
DNMT1:				
Maintenance probability	0.99 (0.97–1.00)	0.98 (0.96–1.00)	0.95 (0.91–0.99)	0.98 (0.95–1.00)
De novo probability	0.02 (0.00–0.05)	0.06 (0.01–0.14)	0.00 (0.00–0.01)	0.01 (0.00–0.02)
Hemi-preference ratio	58 ( 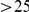 )	15 ( 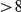 )	628 ( 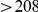 )	94 ( 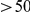 )
Average association level				
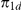	0.99 (0.98–1.00)	0.98 (0.96–0.99)	0.96 (0.89–1.00)	0.99 (0.96–1.00)
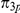	0.02 (0.00–0.05)	0.04 (0.00–0.10)	0.01 (0.00–0.02)	0.01 (0.00–0.02)
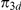	0.05 (0.01–0.08)	0.05 (0.01–0.12)	0.00 (0.00–0.02)	0.01 (0.00–0.03)
Measurement error				
probability ( 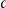 )	0.02 (0.01–0.03)	0.02 (0.01–0.03)	0.02 (0.01–0.05)	0.02 (0.01–0.04)

Medians and 80% credible intervals (CIs) of the posterior distribution of the parameters under our HMM are reported. The lower and upper limit of the 80% CI represent the 10- and 90-percentile, respectively. For hemi-preference ratios, one-sided 80% CIs are reported; that is, the lower limit is the 20-percentile of the distribution. Measurement error probability 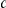 refers specifically to the inappropriate bisulfite conversion probability per CpG per strand (see Materials and Methods for detail). Except for mean association lengths and hemi-preference ratios, estimates reported here were obtained under a uniform (0,1) prior. Entries with an 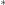 are sensitive to the choice of the prior distribution; in other words, the data are less informative about these parameters.

Recall that our HMM also allows that DNMT1, when associated with the DNA, adds a methyl group to the daughter strand with probability 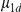 if the parent strand is methylated, and with probability 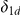 if the parent strand is unmethylated. We estimate 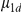 to be close to 1 at both loci (Table 2) and 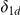 to be just a few percent, with the median being 0.02 at 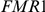 and 0.06 at 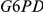 (Table 2), consistent with a very low (not excluding 0) level of de novo activity by DNMT1. These estimates indicate that DNMT1 acting in vivo has a strong preference for hemi-methylated CpG dyads over unmethylated dyads. Our estimates of the hemi-preference ratio 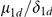 (58 for 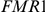 and 15 for 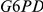) fall within the aforementioned range of estimates obtained in vitro (2–200-fold), but the posterior distributions exclude the lower end of this range (Supplementary Figures 3 and 4 in Materials S1). Note that, because the data do not exclude very small values for 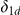, they also cannot exclude extremely large values for the hemi-preference ratio. For this reason Table 2 gives 80% lower confidence bounds, but not upper bounds for this quantity.

### Low association level of in vivo human DNMT3s at two Xi-linked loci

At both Xi-linked loci, we estimated that the average frequency of association (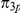 and 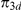) of the DNMT3s is at most a few percent, if non-zero, on either the post-replication parent strand or the daughter strand (Table 2). Such a low average level of association is consistent with a low reassociating probability 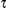 for the DNMT3s: the estimated reassociating probability of the DNMT3s is also not much different from 0 on either strand (Table 2). Because only a limited number of observed methylation events could be attributed to the DNMT3s, there is not much information regarding their processivity or hemi-preference ratio. The posterior distributions of the dissociating probabilities 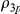 and 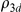 of the DNMT3s are not much different from the uniform prior distribution we assumed (Figure 4(A2)–(A3)), indicating that the data were not informative enough to alter this prior. Thus, these results suggest that the DNMT3s were not very active during the process the observed methylation patterns were formed, and that our data at the two Xi-linked loci are not informative about the processivity, or the lack thereof, of the DNMT3s. Moreover, when we estimated, with additional constraints in the HMM, the hemi-preference ratio for the DNMT3s from the 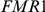 data (Materials S1), we found that its posterior distribution is essentially the same as its prior (Supplementary Figure 6 in Materials S1), which indicates that our data are uninformative also about the preference ratio of the DNMT3s.

### Behavior of in vivo human methyltransferases at autosomal locus 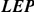


Compared with the two Xi-linked loci, the human 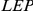 promoter region is much less methylated and is tissue-specific in the data considered here: the overall methylation density is 18% in the adipose tissues (fat) and 42% in the peripheral blood leukocytes (blood), much lower than 85% at 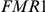 and 82% at 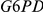. However, most of the methylated CpG dyads are fully methylated: the percentages of fully methylated and hemimethylated dyads are only 16% and 4%, respectively, in fat, and 40% and 5%, respectively, in blood (Table 1). These two 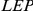 data sets also contain only a few short runs of hemimethylated dyads (Table 1). Our analysis of these data shows that, although the 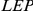 promoter region is sparsely methylated in these tissues, DNMT1 still plays the major role and the rate of maintenance methylation is close to 1 (Table 1; Supplementary Figures 7 and 8 in Materials S1). However, the data are uninformative about what types of process (processive or multiple dissocation-reassociation events) gave rise to this high maintenance methylation rate.

Specifically, the 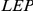 data are uninformative about the dissociating probability 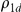 and reassociating probability 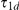 of DNMT1 in fat and blood, as the posterior distributions of both parameters cover the entire support of (0,1) (Table 2; Figure 4(B1); Supplementary Figures 7 and 8 in Materials S1). Furthermore, we estimated the maintenance activity probability of DNMT1 to be very close to 1 and its de novo activity probability to be very close to 0 (Table 2; also see Supplementary Figures 7 and 8 in Materials S1). Thus, the estimated hemi-preference ratio of DNMT1 at 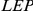 is essentially consistent with that at the Xi-linked loci: this ratio is significantly higher than 1, supporting a preference for hemimethylated CpG sites (Table 2). The median and lower-bound estimates of this ratio at the 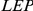 locus is much wider in fat (80% CI: 157–3536) than that in blood, which again suggests that the estimation of this ratio is sensitive to the estimation of the de novo activity probability of DNMT1, and that this ratio can be much higher than the values available in existing literature.

Similar to the 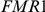 and 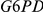 data, the 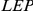 data are also uninformative about the DNMT3s on either strand: the posterior distributions of the dissociating probabilities 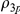 and 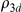 are not substantially different from the uniform prior distribution (Table 2; Figure 4(B2)–(B3)). This uninformativeness may have stemmed also from a low level of enzyme activities: the average association frequencies 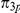 and 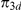 are not substantially different from 0.

### Estimates and impact of measurement errors

Bisulfite conversion used in the experiment can give rise to two types of measurement errors [2], [26] (also see Materials and Methods for details on how we define and incorporate these errors in our analysis). In all the analyses here, we fixed the probability of failure of bisulfite conversion, 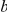, to be 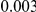 as in our previous analysis [2]. We estimated the probability of inappropriate bisulfite conversion, 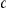, by taking advantage of the result that this probability has little variation across CpGs in our data set [2]. Our estimates for 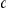 are essentially the same in all the data sets, with the posterior median being 2% and narrow 80% CIs (Table 2; also see Supplementary Figure 9 in Materials S1 for the posterior distribution of 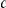 estimated for the 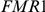 locus). This appreciable error rate is expected under the low-molarity bisulfite-conversion protocol [26] used to collect our data. Note that the results on processivity and substrate preference given above are robust to different assumptions on the measurement error rates: indeed, setting the error rates to be 0 did not qualitatively impact our inference of the hemi-preference ratio or processivity, except producing a slight reduction in the estimated hemi-preference ratio (Supplementary Figure 10 and Table 1 in Materials S1).

Another source of possible measurement errors is PCR crossover, which can occur during PCR amplification with probability less than 1% per molecule [27], leading to ascertained patterns that are hybrids of two molecules [27]. A crossover between one densely methylated and one sparsely methylated molecule may produce a methylation pattern with one of its ends being mostly hemimethylated dyads, and could affect statistical inference on processivity. Take the 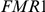 data for example. The probability of having at least 1 of 169 patterns produced by a single crossover is 80% under a binomial distribution. Therefore, pattern #82 from this data set (Figure 2), one of the four most informative patterns and the only pattern with a long run of hemimethylated dyads at an end, may have arisen from a crossover. The other three patterns in Figure 2 have runs of hemimethylated dyads in the middle of the pattern. If these runs were due to crossovers, two events would have had to occur for each molecule. The probability of having at least 1 out of 169 patterns produced by two crossovers is at most 2%. Removing pattern #82 and re-analyzing the rest of the 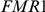 data produced results nearly identical to those for the complete data set (Supplementary Figure 11 in Materials S1). We conclude that PCR crossover errors likely have a negligible impact on our analysis.

### Strong processivity of in vitro mouse DNMT1

**Figure 5:**
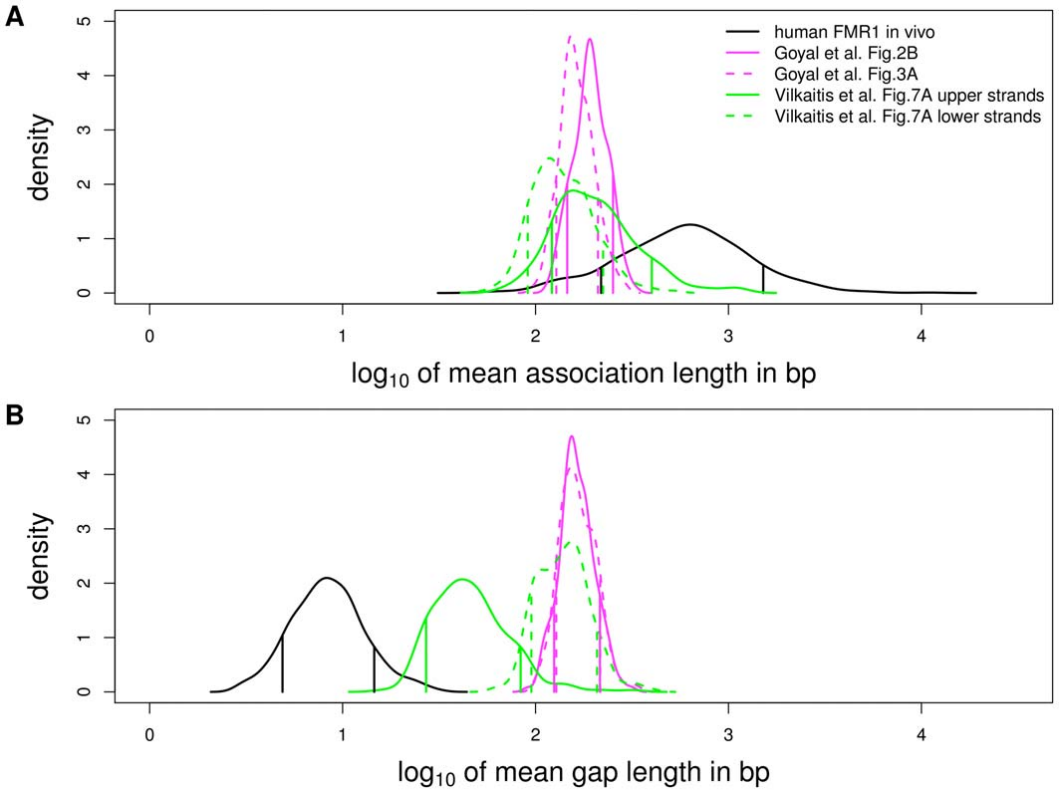
Estimated processivity and non-association tract lengths for human DNMT1 in vivo and mouse DNMT1 in vitro under our HMM. Each curve is the posterior distribution of the (**A**) mean association (processivity) and (**B**) non-association tract lengths on the 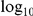 scale. Vertical lines indicate the boundaries of the 80% CIs. Black curves indicate estimates from our in vivo human *FMR1* data. Magenta and green curves are based on our re-analysis of the in vitro mouse DNMT1 data in Goyal et al. [11] and Vilkaitis et al. [10] (Supplementary Figure 12 in Materials S1).

Most previous studies of DNTM1 were conducted in vitro and investigated mouse DNMT1 [10], [11], [28], [29]. These studies did not consider bisulfite-conversion errors, nor did they distinguish genuine processivity from multiple dissocation-reassociation events at consecutive sites. As explained earlier, these data, containing long runs of as many as 20 hemimethylated dyads, suggest a high level of processivity. We re-analyzed double-stranded methylation patterns from Goyal et al. [11] and Vilkaitis et al. [10] under our HMM, setting the association levels of the DNMT3s to 0, and obtained estimates for the processivity of purified mouse DNMT1 acting in vitro (Supplementary Figure 12 in Materials S1; estimates summarized in Supplementary Table 2 in Materials S1). Consistent with the more descriptive analyses in Goyal et al. [11] and Vilkaitis et al. [10], our statistical analyses also estimated a high level of processivity from these in vitro data on mouse DNMT1, with narrow 80% CIs indicating strong evidence from the data (Figure 5).

Although both in vivo and in vitro data show strong levels of processivity, there are noticeable quantitative differences between the two sets of estimates: our estimates from these published in vitro data suggest a shorter mean association tract length (Figures 4 and 5A) and a much longer mean non-association tract length (Figure 5B) than do our estimates of human DNMT1 in vivo (see numeric summaries in Table 2 here and Supplementary Table 2 in Materials S1). These disparities could be due to differences between human DNMT1 and mouse DNMT1, as well as to differences among the experimental conditions in these studies. Disparities could also result from differences between in vivo and in vitro conditions, indicating a role for the replication machinery in modulating enzyme activities. Inferences here and elsewhere [10]–[14] suggest that the replication machinery is not essential for preserving the association of maintenance methyltranferases with the DNA once they are bound. The replication machinery may, however, play a role in keeping unbound DNMT1 poised to re-associate with DNA. This could explain the much shorter non-association tract length and the longer stretches of processive activity inferred here from patterns established in vivo.

## Discussion

In this article, we have developed a novel hidden Markov model to infer complex methyltransferase activities from double-strand methylation patterns established in single molecules. This model complements our earlier model [2] that focuses on estimating the CpG site-specific rates of methylation events, regardless of the methyltransferases. Under our HMM, we can estimate reassociating and dissociating probabilities of the enzymes, as well as probabilities of maintenance and de novo activities, inferring association/non-association tract lengths and hemi-preference ratio. Whereas it is possible that the DNMTs may carry out methylation activities in multiple passes during a round of DNA replication, the processivity our HMM infers here is effectively the “cumulative” processivity. It is also unclear how to incorporate multiple passes of methylation with an unknown number of passes into the statistical modeling. Our model is applicable to in vivo data for which possibly both DNMT1 and the DNMT3s were at work. It is also applicable to in vitro data in which only one type of methyltransferase was present. Since the core of our implementation of the inference of our HMM is the standard forward-backward algorithm, the computing time needed for analyzing these data is linearly proportional to the number of patterns and to the number of CpG sites.

Applying our model to four in vivo human data sets collected at three loci, we find strong evidence for a high level of processivity of DNMT1 at two Xi-linked loci, with the mean association tract lengths being a few hundred to a few thousand bp, whereas the methylation patterns at the autosomal locus 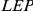 are not informative about processivity. Due to a limited number of loci studied, it is unclear whether the strong processivity of DNMT1 is associated only with the inactive X chromosome. Additionally, the 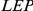 locus may not be representative of autosomal loci, because the data were derived from tissue that is composed of different types (adipose tissue contains adipocyte precursors, blood vessels, and stromal cells besides the mature adipocytes). Although the methylation patterns at the 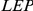 locus have different densities in the two tissue types, our analysis shows that most of the methylation events at this locus are the maintenance methylation activities due to DNMT1 in both tissue types. At all loci examined here DNMT1 showed a strong preference for hemi-methylated CpG sites over unmethylated sites (point estimates ranged from 15 to 628; obtaining precise estimates is difficult because the denominator of the hemi-preference ratio is the probability of de novo methylation events, and these events are rare in our in vivo data dominated by maintenance methylation events).

Our analysis of in vivo data suggests low contributions from the DNMT3s in these in vivo somatic cells. To study the properties of the DNMT3s, an alternative is to analyze double-stranded methylation patterns from in vitro experiments. Such data are indeed available at least for DNMT3A [30], [31]. However, structure analysis suggested that DNMT3A may form a tetramer with DNMT3L, in the form of DNMT3L-DNMT3A-DNMT3A-DNMT3L, which may bind to more than one CpG in a single binding event [32]. We (AQF and MS) are currently extending our HMM to allow for such possibility and carrying out additional analysis for these DNMT3A data in separate work.

## Materials and Methods

### Additional details of the Hidden Markov model

#### Emission probabilities for modeling activities of methyltransferases associated with. DNA

Consider the 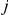-th CpG site on the 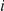-th double-stranded methylation pattern. Let 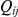 be the methylation state of the post-replication parent CpG at this site and 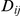 be that of the daughter CpG. Also let 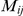, 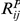 and 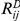 be the association or non-association states of the Markov process of DNMT1 at the daughter CpG, and of the DNMT3s at the parent CpG and the daughter CpG, respectively. The emission probabilities, given as 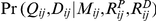, are conditional probabilities and computed for the 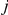-th site as in Table 3.

**Table 3:** Emission probabilities of the HMM.

	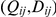			
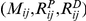	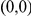	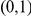	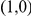	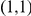
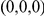	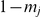	0	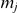	0
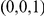	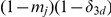	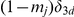	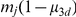	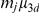
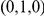	0	0	1	0
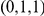	0	0	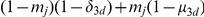	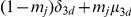
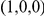	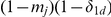	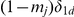	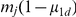	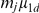
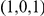	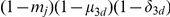	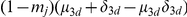	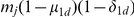	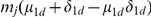
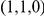	0	0	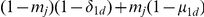	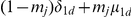
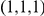	0	0	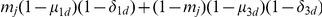	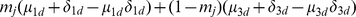

Subscript 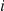 denotes the 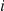-th methylation pattern, and 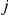 the 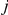-th CpG site. Note that 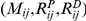 denotes the association (1) or non-association (0) state of DNMT1, parent-strand DNMT3s, and daughter-strand DNMT3s, respectively. Also, 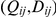 denotes the methylated (1) or unmethylated (0) state of CpG on the parent strand and daughter strand, respectively. Additionally, 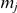 denotes the probability of the 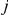-th CpG site being methylated before DNA replication, which is equivalent to the methylation density of the 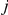-th site. 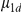 and 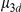 are the probability of the maintenance activity of DNMT1 and the DNMT3s, respectively, at associated daughter-strand CpG, whereas 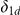 and 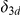 are that of the de novo activity of DNMT1 and the DNMT3s, respectively. The measurement error rates (see Materials and Methods and Table 4) are assumed to be 0 here. This HMM allows for estimation of the hemi-preference ratio for both DNMT1 and the DNMT3s, although additional constraints are needed for this simultaneous estimation. See text for details.

Each entry in Table 3 sums over two states: methylation and no methylation at the pre-replication parent CpG, with probability 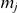 and 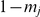, respectively. For example, consider the following entry near the bottom right of Table 3, 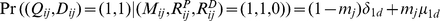. In this case, DNMT1 and the DNMT3s are both associated with the CpG dyad, with the former at the daughter CpG (i.e., 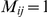) and the latter at the parent CpG (i.e., 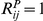, 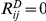). Either of two events occurred for the formation of the observed fully methylated CpG dyad (i.e., 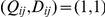): (i) DNMT1 carried out a maintenance methylation event on the daughter CpG of the dyad where the parent CpG had been methylated before replication. This event has probability 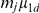; or (ii) the dyad, unmethylated before replication with probability 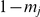, became methylated de novo on the daughter strand by DNMT1 with probability 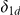 and on the parent strand by the DNMT3s with probability 1. This double de novo methylation event has total probability 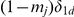.

We made two assumptions in the calculation of the emission probabilities: (i) measurement errors did not occur in the collection of our data; relaxation of this assumption to incorporate error is described below; and (ii) there is no active removal of methyl groups on the parent strand when DNA is replicated [2], [21], [23], [33], which means that a CpG methylated before replication remains methylated after replication. Although active removal of methyl groups has been reported during early development, in cancer cells (see [34] for review), and for transcriptionally active loci under perturbation [35], [36], this phenomenon seems uncommon, if it occurs at all, in somatic cells in normal individuals, which are the cell types we study here.

To distinguish between the DNMT1 process and the DNMT3 process on the daughter strand, in the simplest version of the HMM we draw on the existing evidence that the two classes of methyltransferases exhibit different substrate preferences [4], [6], [7], [18], [37], [38]. When the DNMT3s are associated with the daughter strand, we assume that they add a methyl group with probability 1 (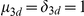) at both hemimethylated and unmethylated CpGs. That is, association is synonymous to methylation for the DNMT3s on the daughter strand. In contrast, when DNMT1 is associated with the daughter strand, we allow it to methylate CpGs at hemimethylated sites and at unmethylated sites with different probabilities 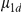 and 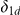, respectively, with the ratio 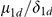 being the hemi-preference ratio. To also estimate the hemi-preference ratio for the DNMT3s on the daughter strand, 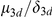, we use a different set of constraints, namely 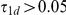 and 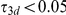 (Materials S1).

#### Incorporating measurement errors due to bisulfite conversion

We consider two types of measurement errors due to bisulfite conversion [2], [26]: failure of bisulfite conversion, which occurs with probability 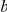 per CpG, and inappropriate bisulfite conversion, which occurs with probability 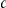 per CpG (see definitions in Table 4). We assume that these errors occur independently across CpGs and DNA strands [2]. Denote 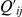 and 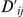 as the observed methylation states at the parent and daughter CpGs, respectively, on the 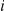-th methylation pattern at the 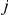-th CpG dyad, with possible measurement error. Emission probabilities accounting for these measurement errors are:
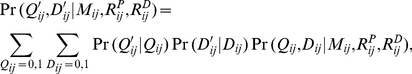
(3)where 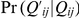 and 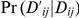 are functions of measurement error probabilities 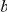 and 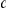 (Table 4), whereas 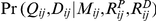 is previously defined as the emission probability without measurement error (Table 3).

**Table 4:** Probabilities of measurement errors due to bisulfite conversion.

		Observed ( 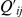 or 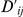 )	
		0	1
Truth ( 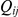 or 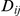 )	0	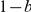	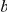
	1	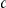	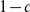

True and observed methylation state of the parent-strand CpG in the 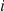-th double-strand methylation pattern at the 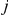-th site are denoted 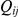 and 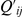, respectively. True and observed methylation states on the daughter-strand CpG are denoted 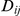 and 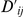, respectively. Additionally, 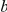 is the probability of failure of bisulfite conversion at a CpG, and 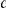 the probability of inappropriate bisulfite conversion.

#### Software implementing the HMM

The models and analyses presented here are implemented in the computer program MethylHMM, which can be downloaded from http://stephenslab.uchicago.edu/software.html. This program includes also all the data sets analyzed here.

### Human in vivo double-stranded methylation data

We used hairpin-bisulfite PCR [21] to collect double-stranded methylation patterns of single molecules from the promoter region of genes 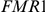 and 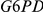, two loci on the inactive X chromosome, and gene 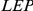 on Chromosome 7. Molecular barcodes and batchstamps were used to help identify and remove contaminant and redundant methylation patterns [22]. To capture the variation in methylation patterns across cells, we collected multiple patterns from each individual sampled (Table: 1). Each pattern (see examples in Figure 2) consists of methylation states at CpG sites on the parent and daughter strands at this locus in an individual cell, with no direct information as to which is the parent strand and which is the daughter. The 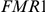 and 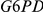 data were collected on the hypermethylated, inactivated X chromosome in somatic lymphocytes from human females. The 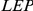 data were collected from fat tissues (abdomen and breast) and peripheral blood leukocytes. The 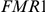 data were previously analyzed in Fu et al. [2] under a different statistical model that assumed the methylation events at different CpG sites to be independent. The 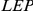 data were publised in Stöger [39]. Features of the loci and summary statistics of the methylation data are presented in Table 1.

## Supporting Information

Materials S1 (PDF)
