# Supplementary material for: Statistical Inference of In Vivo Properties of Human DNA Methyltransferases from Double-Stranded Methylation Patterns
Source: PLoS One. 2012 Mar 19;7(3):e32225. doi: 10.1371/journal.pone.0032225 (PMC3307717; doi:10.1371/journal.pone.0032225)
Supplement: Materials S1 — (PDF) [file pone.0032225.s001.pdf]

Supporting Information for "Statistical Inference of In Vivo Properties of Human DNA Methyltransferases from Double-Stranded Methylation Patterns"

Audrey Q. Fu[1,∗,†], Diane P. Genereux[2], Alice F. Burden[2], Reinhard Stöger[3], Charles D. Laird[2], Matthew Stephens[4]

1 Department of Physiology, Development & Neuroscience, and Cambridge Systems Biology Centre, University of Cambridge, Cambridge, United Kingdom

2 Department of Biology, University of Washington, Seattle, Washington, United States of America

3 School of Biosciences, University of Nottingham, Sutton Bonington Campus, Leicestershire, United Kingdom

4 Departments of Human Genetics and Statistics, University of Chicago, Chicago, Illinois, United States of America

∗ E-mail: audreyqyfu@uchicago.edu

† Current address: Department of Human Genetics, University of Chicago, Chicago, Illinois, United States of America

This document contains supplementary figures, tables, and text. Text in this document contains details of alternative hidden Markov models, and our Bayesian inference approach, algorithms, and model checking.

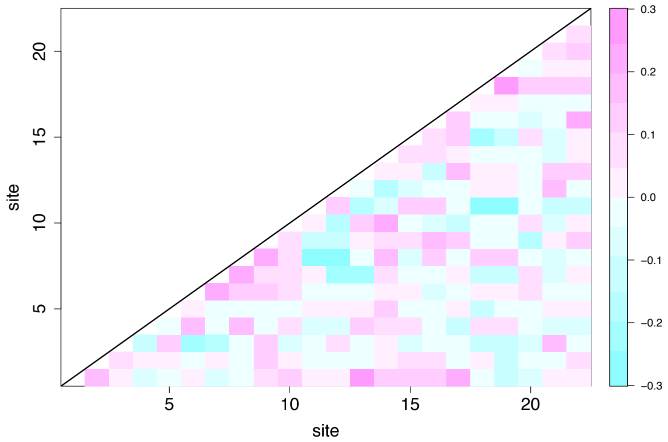

Supplementary Figure 1. Heat map of pairwise correlations of hemimethylated CpG dyads in the *FMR1* data. To compute the correlations, we represented each dyad in the data as being in one of three states: hemimethylated with the methyl group on the top strand (denoted by 1), hemimethylated with the methyl group on the bottom strand (denoted by −1), and fully- or un-methylated (both denoted by 0). We then calculated Pearson correlation for each pair of CpG sites, and represented each correlation as a rectangle in the heat map. The rectangles immediately off the diagonal show, on average, relatively high correlations between adjacent sites, indicating short-range dependence. A permutation test (permuting CpG sites) was carried out for the average of correlations immediately off the diagonal and confirmed this dependence with a significant p value of 8×10^{-4}.

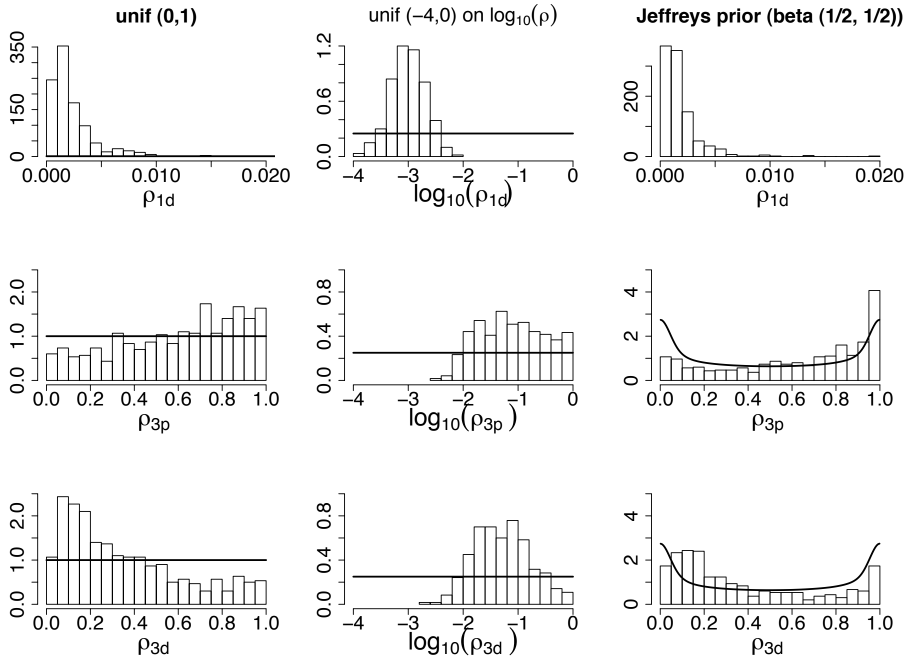

Supplementary Figure 2. Robustness of estimates of dissociating probability ρ to different prior distributions for the *FMR1* data. Dark lines, not noticeable due to range on the vertical axis in some plots, indicate the density functions of prior distributions. For ρ_{1d} of DNMT1 (**Top Row**), its estimation is robust in the sense that the estimates do not vary appreciably under different priors. For ρ_{3p} (**Middle Row**) and ρ_{3d} (**Bottom Row**) of the DNMT3s, the posterior distributions are essentially the same as the priors, indicating that the data are not very informative for these values. In the bottom two plots of the middle column, the data are not informative enough to produce estimates as small as below -3 on $\log_{10}$ scale. See Sec. 2 in Supplementary Text for details on the priors.

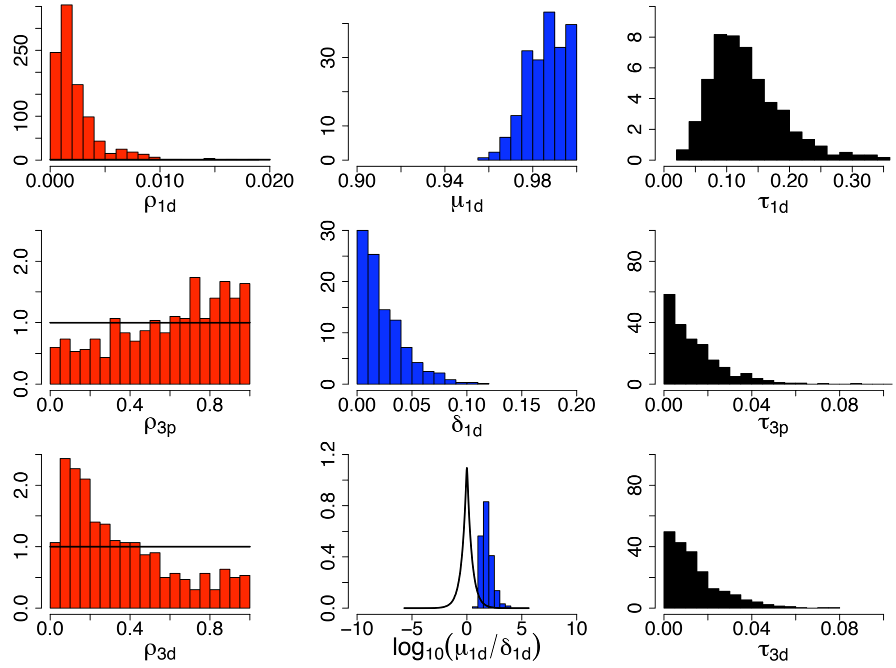

Supplementary Figure 3. Posterior distributions of key parameters under the main HMM for the *FMR1* data. Figure 4 of the main text is shown in the left column, with scales different from those in Figure 4(A1)-(A3) in the main text, for ease in comparison with other similar supplementary figures. Left and right columns show dissociating probability ρ (**Left Column**) and reassociating probability τ (**Right Column**) for DNMT1 on the daughter strand (**Top Row**), the DNMT3s on the parent strand (**Middle Row**), and the DNMT3s on the daughter strand (**Bottom Row**). Middle column shows the maintenance (**Top**) and de novo (**Middle**) methylation probabilities for DNMT1 when associated with DNA, as well as its hemi-preference ratio (on the $\log_{10}$ scale; **Bottom**). Dark lines, where shown, indicate the density of prior distributions assigned to the parameters. See Sec. 2 in Supplementary Text for details on the priors.

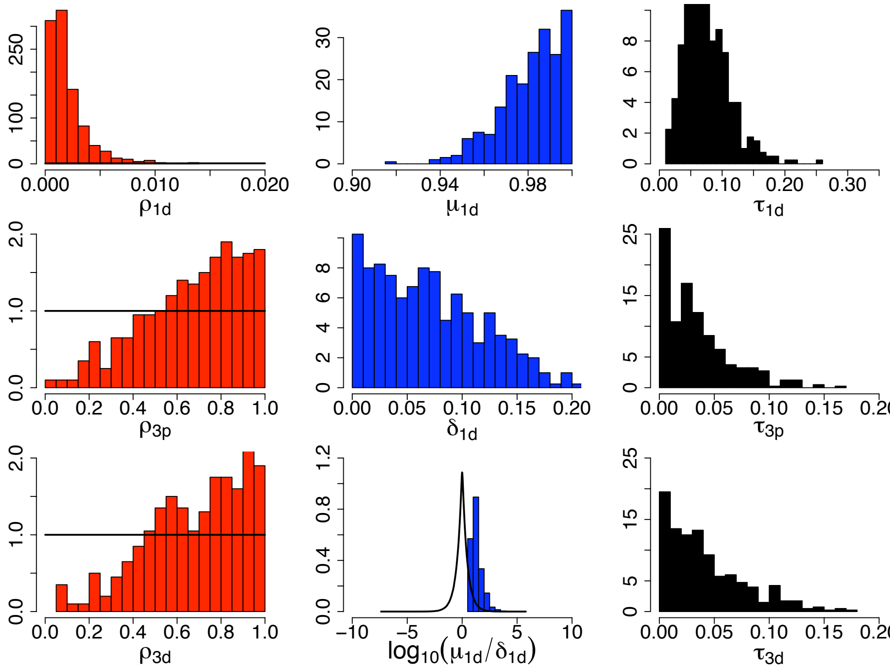

Supplementary Figure 4. Posterior distributions of key parameters under our HMM for the *G6PD* data. Left and right columns show dissociating probability ρ (**Left Column**) and reassociating probability τ (**Right Column**) for DNMT1 on the daughter strand (**Top Row**), the DNMT3s on the parent strand (**Middle Row**), and the DNMT3s on the daughter strand (**Bottom Row**). Middle column shows the maintenance (**Top**) and de novo (**Middle**) methylation probabilities for DNMT1 when associated with DNA, as well as its hemi-preference ratio (on the $\log_{10}$ scale; **Bottom**). Dark lines, where shown, indicate the density of prior distributions assigned to the parameters.

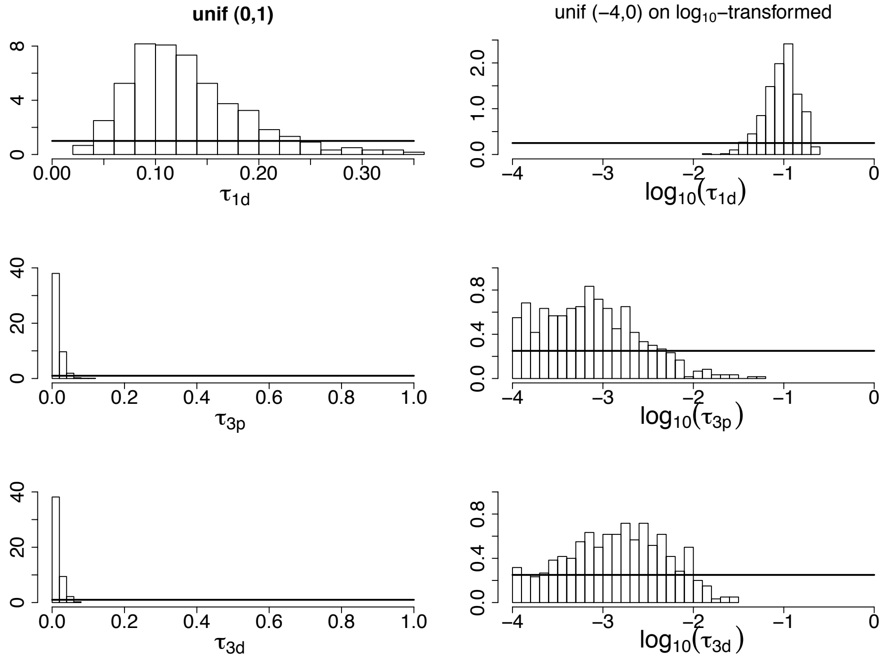

Supplementary Figure 5. Robustness of estimates of reassociating probability τ to different prior distributions for the *FMR1* data. Dark lines indicate the density functions of prior distributions. The posterior distribution for any τ is essentially the same under the uniform$(0, 1)$ (**Left Column**) and $\log_{10}$ uniform$(-4, 0)$ (**Right Column**) priors. See Sec. 2 of Supplementary Text for details on the priors.

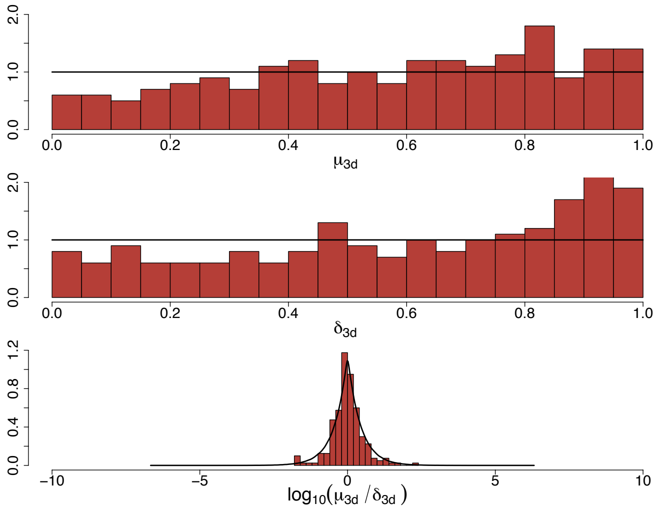

Supplementary Figure 6. Estimation of hemi-preference ratio for the DNMT3s on the daughter strand under the variant HMM for the *FMR1* data. This variant HMM allows for the possibility that DNMT3s on the daughter strand have different probabilities of adding methyl group to hemi- as compared with unmethylated dyads. This contrasts with the main HMM, which assumes equal probabilities for these two types of events. Posterior distributions are shown for maintenance-type (**Top**) and de novo-type (**Middle**) methylation probabilities, and hemi-preference ratio (**Bottom**), of the DNMT3s on the daughter strand. Dark lines show density functions of the priors. The *FMR1* data are not informative about these parameters. Inference of other parameters is the same as under the main HMM. See Supplementary Text for details of this variant model.

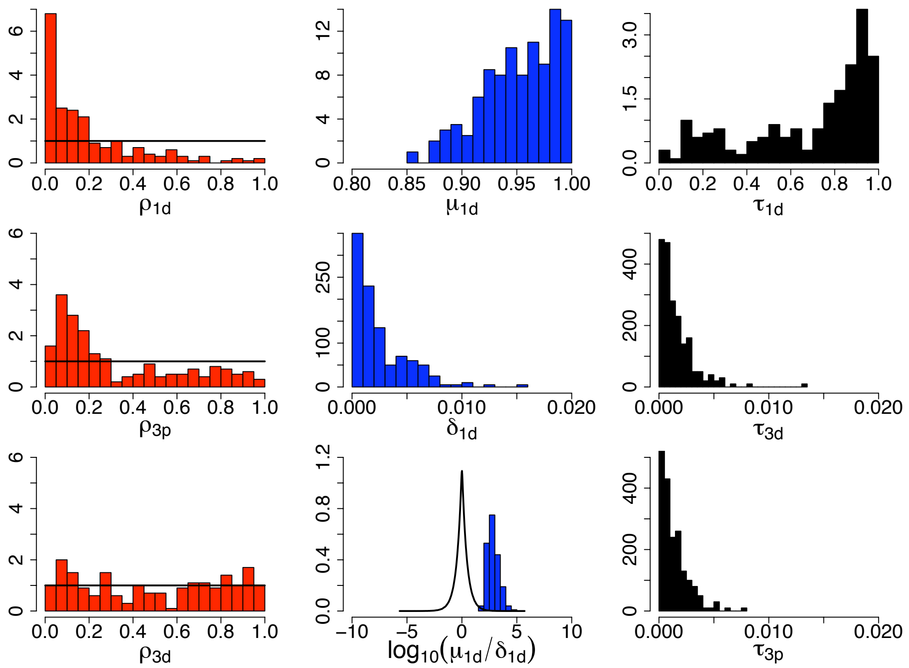

Supplementary Figure 7. Posterior distributions of key parameters under our HMM for the *LEP* data from the adipose tissues (fat). Left and right columns show dissociating probability ρ (**Left Column**) and reassociating probability τ (**Right Column**) for DNMT1 on the daughter strand (**Top Row**), the DNMT3s on the parent strand (**Middle Row**), and the DNMT3s on the daughter strand (**Bottom Row**). Middle column shows the maintenance (**Top**) and de novo (**Middle**) methylation probabilities for DNMT1 when associated with DNA, as well as its hemi-preference ratio (on the $\log_{10}$ scale; **Bottom**). Dark lines, where shown, indicate the density of prior distributions assigned to the parameters.

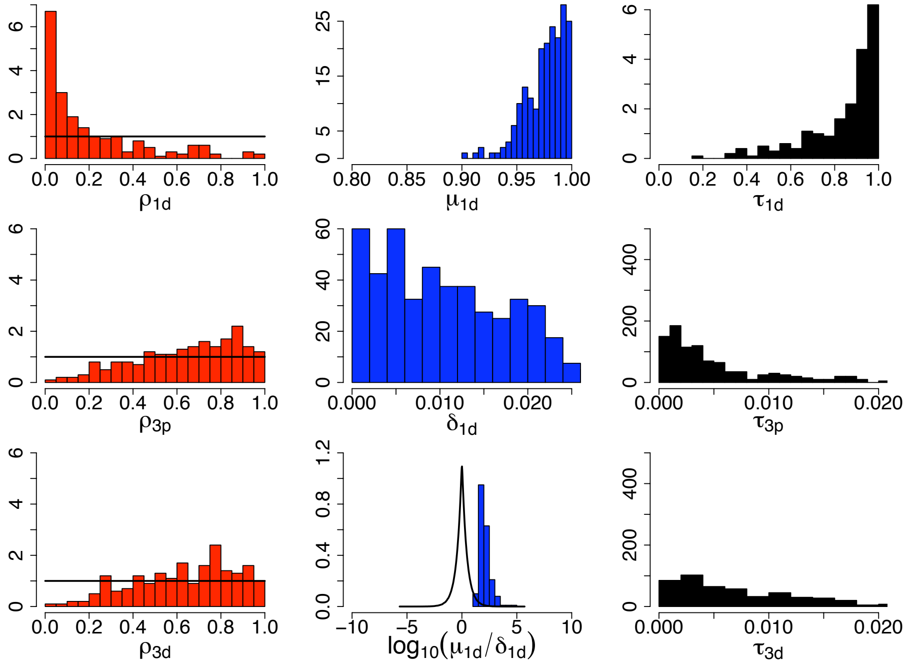

Supplementary Figure 8. Posterior distributions of key parameters under our HMM for the *LEP* data from peripheral blood leukocytes (blood). Left and right columns show dissociating probability ρ (**Left Column**) and reassociating probability τ (**Right Column**) for DNMT1 on the daughter strand (**Top Row**), the DNMT3s on the parent strand (**Middle Row**), and the DNMT3s on the daughter strand (**Bottom Row**). Middle column shows the maintenance (**Top**) and de novo (**Middle**) methylation probabilities for DNMT1 when associated with DNA, as well as its hemi-preference ratio (on the $\log_{10}$ scale; **Bottom**). Dark lines, where shown, indicate the density of prior distributions assigned to the parameters.

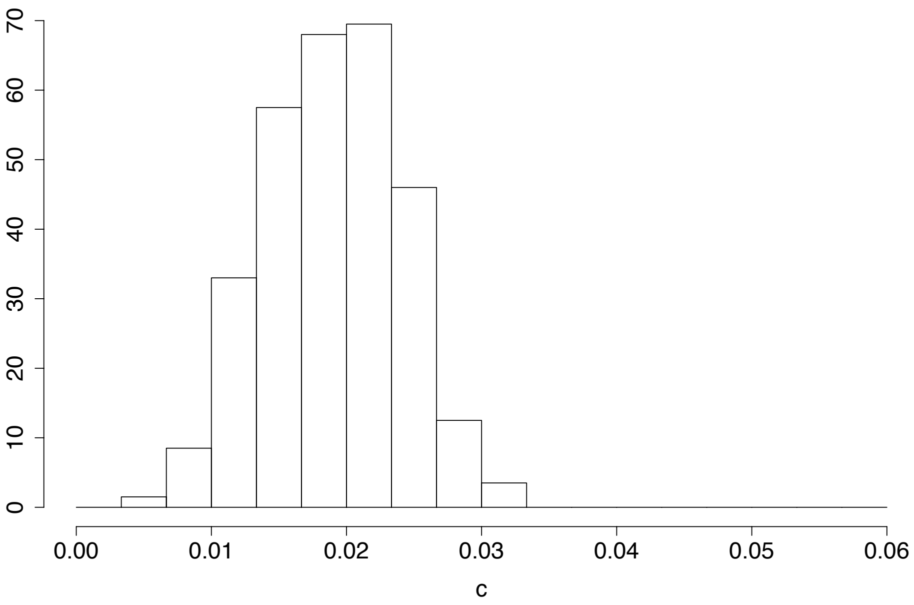

Supplementary Figure 9. Posterior distribution of the rate of measurement error due to inappropriate bisulfite conversion, c, under the main HMM for the *FMR1* data.

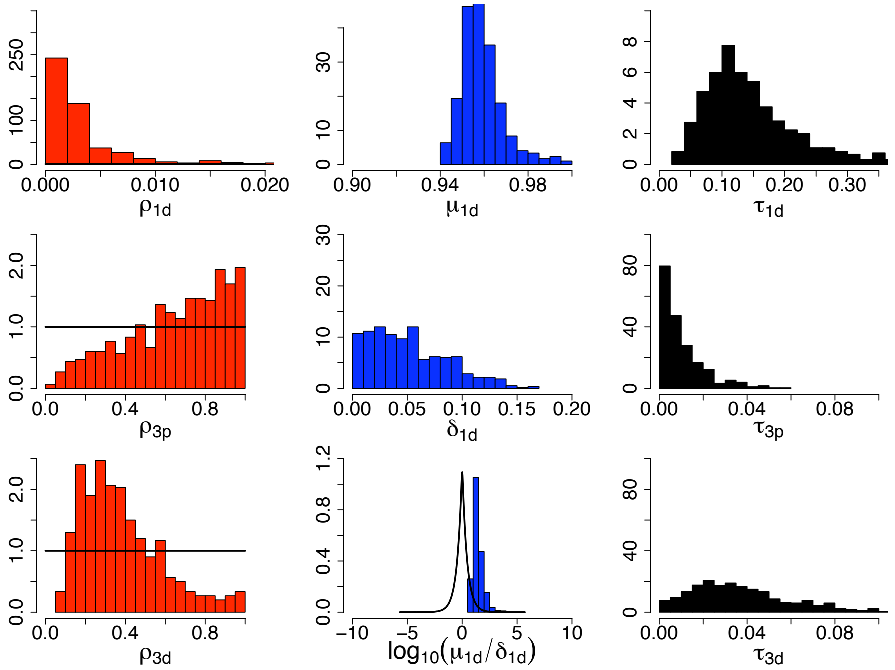

Supplementary Figure 10. Impact of measurement errors due to bisulfite conversion on estimation under our HMM for the *FMR1* data. Format of figure is the same as for Supplementary Figure 3. We re-ran the main HMM on the *FMR1* data with the inappropriate conversion error rate c fixed to 0. The other measurement error rate b is still fixed to be 0.003 (setting $b = 0$ did not produce noticeably different results). Comparison with Supplementary Figure 3 suggests that (i) the inappropriate bisulfite conversion error, although having a significantly non-zero rate, does not affect the inference of processivity. Estimates of ρ are qualitatively the same whether this error is accounted for or not; (ii) not accounting for this error inflates δ_{1d} and deflates μ_{1d}, which leads to a slightly reduced hemi-preference ratio (see numerical summaries in Supplementary Table 1). This is because, under this $c = 0$ model, some observed hemimethylated dyads, although due to genuine measurement errors, now have to be explained by DNMT1 activities; (iii) not accounting for this error also inflates τ_{3d} and thus the overall inferred activity level of the DNMT3s (Supplementary Table 1). Activities of DNMT3s on the daughter strand are invoked under the $c = 0$ model to explain some hemimethylated dyads that are due to genuine measurement errors.

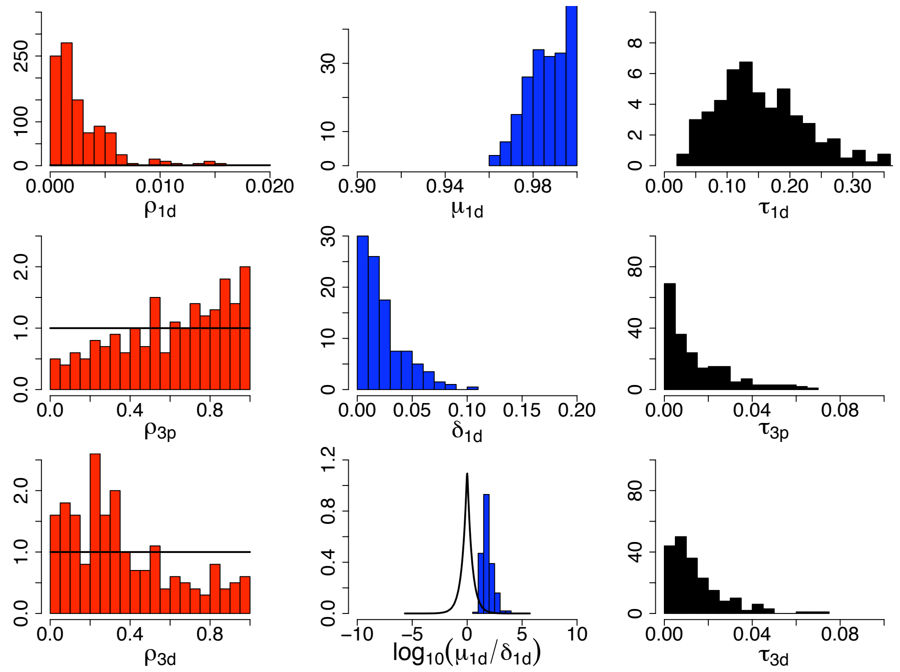

Supplementary Figure 11. Impact of PCR crossover error on inference for the *FMR1* data. Format of figure is the same as for Supplementary Figure 3. We removed pattern #82 (see Figure 2 in the main text), which is a plausible product of PCR crossover, and re-ran the main HMM to obtain posterior distributions of the key parameters. Results are essentially the same as those in Supplementary Figure 3, suggesting that PCR crossover errors are likely to have a negligible impact on the inference.

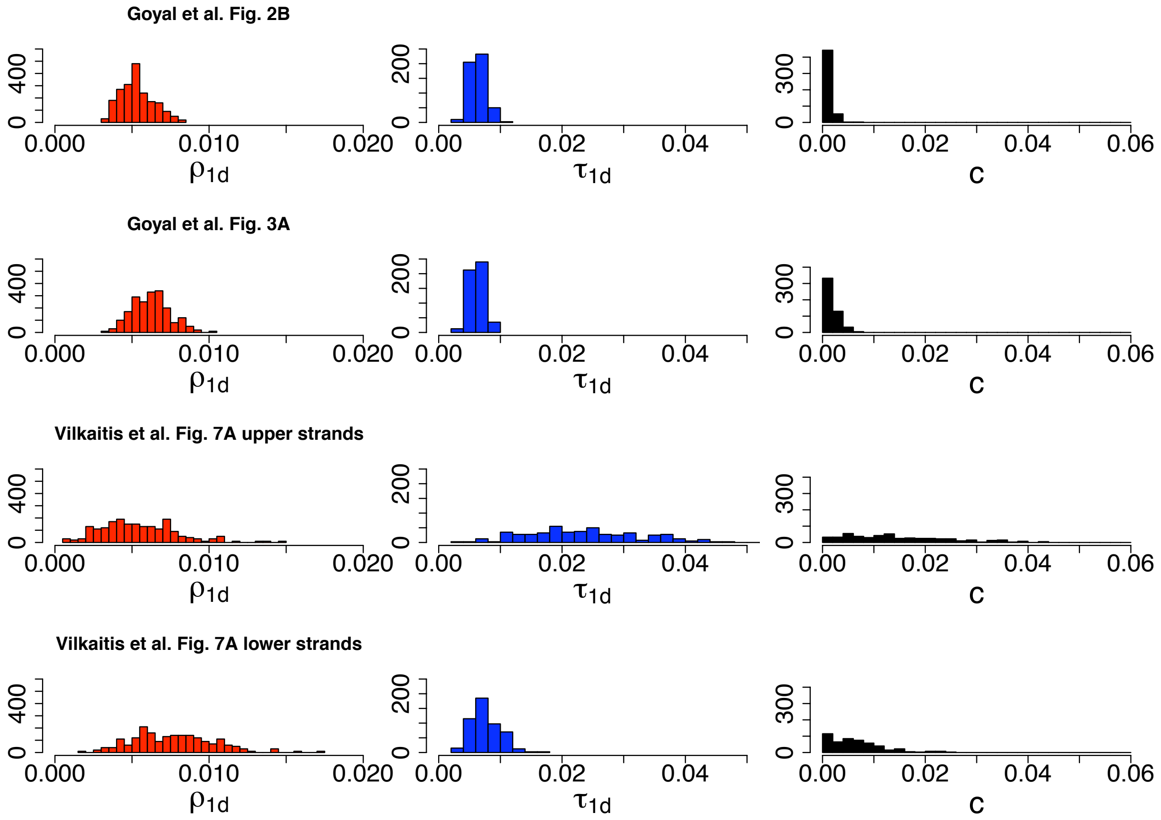

Supplementary Figure 12. Re-analysis of published in vitro data on mouse DNMT1. Published data are taken from figures of Goyal et al. [1] and Vilkaitis et al. [2], as indicated in the figure. Positions in bp of the CpG sites for data from [1] are for Substrate 1 as given in their Supplemental Information. Positions in bp of the CpG sites for data from [2] are as in Figure 2 of their main text. Each row shows estimates from a different published data set using our modified HMM model (see Sec. 1.2 in Supplementary Text for details). Key parameters here are dissociating probability ρ_{1d} (**Left Column**), reassociating probability τ_{1d} (**Middle Column**), and bisulfite-conversion error rate c (**Right Column**).

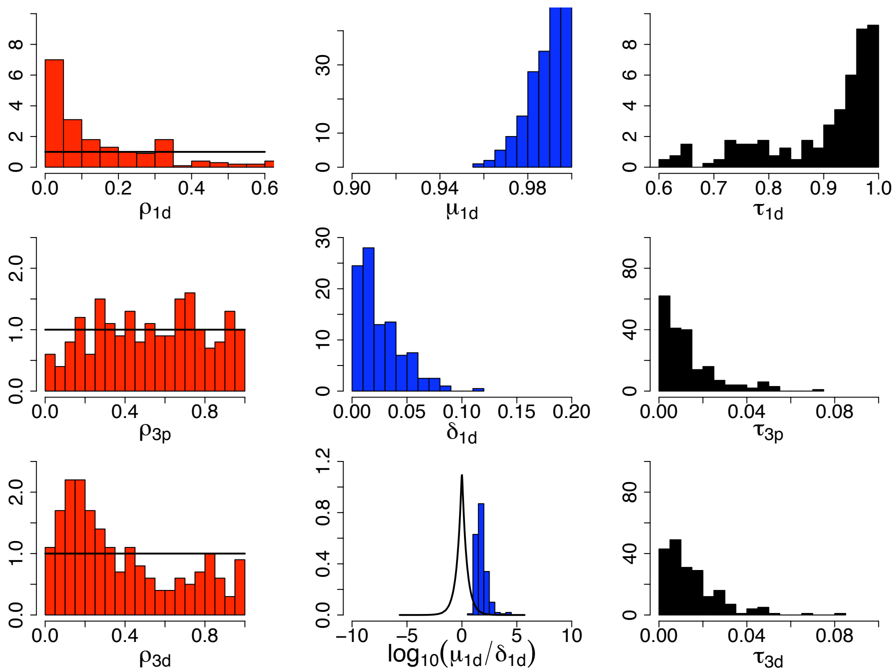

Supplementary Figure 13. Impact of the four most informative patterns in the *FMR1* data (see Figure 2 in the main text) on parameter inference. Format of figure is the same as that of Supplementary Figure 3, although axes ranges differ for some plots. We removed the four most informative patterns from our *FMR1* data and re-ran the main HMM on the remaining *FMR1* data. Posterior distributions of key parameters are displayed. Comparison with Supplementary Figure 3 suggests that (i) the data, even without the four patterns, still provide strong evidence for processivity in DNMT1, although the inferred level of processivity is reduced. Specifically, dissociating probability ρ_{1d} is now estimated to be 0.09 (80% CI: 0.005-0.56), corresponding to a mean association tract length of 11 bp (80%: 2-219 bp); (ii) the data without the four patterns are also informative about other aspects of DNMT1, such as the overall association level (estimated 0.99 with 80% CI of 0.98-1.00, based on estimates of ρ_{1d} and τ_{1d}), the maintenance and de novo activity rates, and the hemi-preference ratio. Estimates of these four parameters are the same as from the complete data; and (iii) reassociating probability τ_{1d} is estimated much higher here than from the complete data (0.12; 80% CI, 0.07-0.20. Supplementary Figure 3). Many of the DNMT1 association events are now interpreted as independent associations at individual CpG sites.

Supplementary Table 1. Impact of bisulfite-conversion measurement errors on estimation under the HMM for the *FMR1* data.

	Estimates from the HMM estimating c	$c = 0$
DNMT1		
Maintenance activity rate μ_{1d}	0.99 (0.97-1.00)	0.96 (0.95-0.97)
De novo activity rate δ_{1d}	0.02 (0.00-0.05)	0.05 (0.01-0.10)
Hemi-preference ratio μ_{1d}/δ_{1d}	58 (Pr{> 25} = 0.9)	21 (Pr{> 10} = 0.9)
DNMT3s on daughter strand		
Reassociating probability τ_{3d}	0.01 (0.00-0.03)	0.03 (0.01-0.07)
Average activity level π_{3d}	0.05 (0.01-0.08)	0.10 (0.04-0.15)

The differences between the results under the main HMM estimating c (Supplementary Figure 3) and that setting $c = 0$ (Supplementary Figure 10) are shown. The other bisulfite-related measurement error rate b is set to 0.003 under both models. A uniform (0,1) prior is assigned to ρ for both models. Unless stated otherwise, the numbers reported here are posterior medians with 80% credible intervals given in parentheses.

Supplementary Table 2. Parameter estimates under our HMM for the in vitro DNMT1 data sets.

	Goyal et al. [1]		Vilkaitis et al. [2]Fig. 7A	
Parameter	Fig. 2B	Fig. 3A	Upper Panel	Lower Panel
Mean association length (bp) of DNMT1	192 (146-252)	161 (128-211)	194 (122-401)	133 (91-209)
Dissociating probability ρ_{1d}	0.005 (0.004-0.007)	0.006 (0.005-0.008)	0.005 (0.002-0.008)	0.008 (0.004-0.011)
Reassociating probability τ_{1d}	0.006 (0.005-0.008)	0.006 (0.005-0.008)	0.023 (0.012-0.037)	0.007 (0.005-0.010)
Average association level π_{1d}	0.54 (0.46-0.61)	0.50 (0.41-0.58)	0.82 (0.73-0.89)	0.49 (0.41-0.61)
Measurement error probability c	0.00 (0.00-0.00)	0.00 (0.00-0.00)	0.01 (0.00-0.03)	0.01 (0.00-0.01)

The numbers reported here are posterior medians with 80% credible intervals given in parentheses.

Supplementary Text

1 Alternative Hidden Markov Models

1.1 HMM to estimate the hemi-preference ratio for the DNMT3s

As mentioned in the main text, our HMM with the emission probability matrix defined in Table 3 in the main text models the DNMT1 process and the DNMT3s process on the daughter strand in the same way. Additional constraints are needed to distinguish the two processes. Our earlier analysis assuming $\mu_{3d} = \delta_{3d} = 1$, where μ_{3d} and δ_{3d} are the maintenance activity probability and the de novo activity probability of the DNMT3s on the daughter strand, respectively, indicated that the reassociating probability of DNMT1, τ_{1d}, is likely to be close to 1, and that of the DNMT3s on the daughter strand, τ_{3d}, is likely to be close to 0. Hence, we imposed constraints $\tau_{1d} > 0.05$ and $\tau_{3d} < 0.05$ when estimating μ_{3d} and δ_{3d}.

1.2 Re-analysis of published in vitro mouse DNMT1 data

Because the DNMT3s were not present in the in vitro experiments published in Vilkaitis et al. [2] and Goyal et al. [1], we fix in the main HMM reassociating probabilities τ_{3p} and τ_{3d} for the DNMT3s to be 0 and dissociating probabilities ρ_{3p} and ρ_{3d} to be 1. We also fix μ_{1d} to be 1 and δ_{1d} to be 0, because the template strand is fully (or nearly fully) methylated in these experiments. Key parameters of interest are then ρ_{1d}, τ_{1d} and measurement error probability c.

2 Bayesian inference under the HMMs

2.1 The likelihood

In a data set of N double-stranded binary methylation patterns at S CpG sites, the i-th pattern is represented as an unordered pair of binary vectors $\{\mathbf{x}_i, \mathbf{y}_i\}$. This is an unordered pair because the hairpin-bisulfite methylation data do not provide information of which strand in each pattern is the parent strand, and which strand is the daugher. Recall that, accounting for measurement errors, the pattern on the parent strand is denoted $\mathbf{Q}'_i$ and that on the daughter strand $\mathbf{D}'_i$. We let $\lambda = \{\tau_{1d}$, τ_{3p}, τ_{3d}, ρ_{1d}, ρ_{3p}, ρ_{3d}, μ_{1d}, δ_{1d}, b, c, m_j, $j = 1, 2, \ldots, S\}$ denote the parameters of interest. The likelihood of observing N double-stranded patterns given λ is

$$L(\lambda; \{\mathbf{x}, \mathbf{y}\}) = \prod_{i=1}^{N} \Pr(\{\mathbf{Q}'_i, \mathbf{D}'_i\} = \{\mathbf{x}_i, \mathbf{y}_i\} | \lambda) \tag{1}$$

$$\propto \prod_{i=1}^{N} \{\Pr((\mathbf{Q}'_i, \mathbf{D}'_i) = (\mathbf{x}_i, \mathbf{y}_i)|\lambda) + \Pr((\mathbf{Q}'_i, \mathbf{D}'_i) = (\mathbf{y}_i, \mathbf{x}_i)|\lambda)\}, \tag{2}$$

where $(\mathbf{x}_i, \mathbf{y}_i)$ and $(\mathbf{y}_i, \mathbf{x}_i)$ in Eq. (2) represent ordered pairs, each corresponding to a strand assignment. Specifically, $(\mathbf{x}_i, \mathbf{y}_i)$ represents that the top strand $\mathbf{x}_i$ is the parent strand $\mathbf{Q}'_i$, whereas the bottom strand $\mathbf{y}_i$ is the daughter strand $\mathbf{D}'_i$; and $(\mathbf{y}_i, \mathbf{x}_i)$ represents that the top strand $\mathbf{x}_i$ is the daughter strand $\mathbf{D}'_i$, whereas the bottom strand $\mathbf{y}_i$ is the parent strand $\mathbf{Q}'_i$. The strand assignment probability

$\Pr\{(\mathbf{Q}_i', \mathbf{D}_i') = (\mathbf{x}_i, \mathbf{y}_i)|\lambda\}$ is computed as follows,

$$\Pr\{(\mathbf{Q}_i', \mathbf{D}_i') = (\mathbf{x}_i, \mathbf{y}_i)|\lambda\} = \sum_{\mathbf{M}_i, \mathbf{R}_i^p, \mathbf{R}_i^d} \Pr\{(\mathbf{Q}_i', \mathbf{D}_i') = (\mathbf{x}_i, \mathbf{y}_i)|\mathbf{M}_i, \mathbf{R}_i^p, \mathbf{R}_i^d, \lambda\} \times \Pr(\mathbf{M}_i|\lambda)\Pr(\mathbf{R}_i^p|\lambda)\Pr(\mathbf{R}_i^d|\lambda), \quad (3)$$

where emission probability $\Pr\{(\mathbf{Q}_i', \mathbf{D}_i') = (\mathbf{x}_i, \mathbf{y}_i)|\mathbf{M}_i, \mathbf{R}_i^p, \mathbf{R}_i^d, \lambda\}$ is defined in Eq. (3) in the main text. We use the standard forward-backward algorithm [3], which is a dynamic programming algorithm, to compute Eq. (3) here. We use the extended Viterbi algorithm (Sec. 4), also a dynamic programming algorithm, to make inference based on Eq. (3). Probability of the other strand assignment, $\Pr((\mathbf{Q}_i', \mathbf{D}_i') = (\mathbf{y}_i, \mathbf{x}_i)|\lambda)$, is defined and calculated similarly. The strand assignment with a higher posterior probability between the two possibilities, calculated using estimates of λ, is displayed in Figures 2 and 3 of the main text.

2.2 Distribution assumptions

Under the Bayesian framework, we assume a prior distribution for each parameter before estimation, and aim to obtain the posterior distributions, instead of just point estimates, for the parameters of interest given the observations and the priors. Differences between the posterior and prior distributions for a parameter reflect the information that the data provide for this parameter. Because of the important role prior distributions play in parameter estimation, we experimented with different priors for key parameters ρ and τ to assess their impact on estimation.

We experimented with three prior distributions for each dissociating probability ρ: (i) uniform $(0, 1)$, which assigns equal probability mass everywhere between 0 and 1. This is the most natural prior for a probability parameter; (ii) beta $(\alpha = 1/2, \beta = 1/2)$, also known as Jeffreys prior for proportions [4], which is a non-informative prior and assigns more probability mass near 0 and 1 than at values in between. For ρ, the two extremes 0 and 1 correspond to strong processivity and no processvity, respectively. Comparing the posterior with this prior would allow us to informally test these two hypotheses; and (iii) uniform $(-4, 0)$ on the $\log_{10}$ scale, which puts more probability mass at values near 0. This prior is particularly useful if processivity seems to exist. It helps confirm the existence and allows a more accurate estimation of processivity.

We investigated the effect of priors (i) and (iii) on reassociating probability τ. Prior (ii) was not used for τ because the the data are fairly informative about τ.

We assigned a uniform $(0, 1)$ prior distribution to the maintenance methylation probability μ_{1d} of DNMT1, the de novo methylation probability δ_{1d} of DNMT1, and the measurement error rate c. This assignment induces a prior distribution on the log-transformed hemi-preference ratio, $\log_{10}(\mu_{1d}/\delta_{1d})$.

To estimate site-specific methylation probability m_j, we took a hierarchical modeling approach as in [5] to reduce complexity. That is, we assumed that all the m_j at S sites arise from a single distribution, beta (r_m, g_m), such that

$$\mathrm{E}(m_j) = r_m, \quad \mathrm{Var}(m_j) = g_m r_m (1 - r_m). \quad (4)$$

As in [5], we assigned a uniform (0,1) prior to mean r_m. We used a uniform $(-4, 0)$ prior for scaled variance $\log_{10}(g_m)$; this prior captures variability in a wide range, and allows us to distinguish among small variations, such as 0.001 and 0.01. We consider the variation among m_j to be low if $\log_{10}(g_m) < -2$, medium if $\log_{10}(g_m)$ is between -2 and -1, and high if above 1.

3 The Markov Chain Monte Carlo (MCMC) procedure

We used a Metropolis-Hastings procedure [6] to update parameters in $\lambda = \{\tau_{1d}, \tau_{3p}, \tau_{3d}, \rho_{1d}, \rho_{3p}, \rho_{3d}, \mu_{1d}, \delta_{1d}, b, c, m_j, j = 1, 2, \ldots, S\}$ based on the likelihood $L(\lambda; \{\mathbf{x}, \mathbf{y}\})$ defined in Eq. 2. Specifically, each

iteration of the MCMC involves the following steps:

Step 1. Updates each of the reassociating probabilities τ and dissociating probabilities ρ.

Step 2. Updates methylation probabilities m_j jointly.

Step 3. Updates the mean parameter r_m.

Step 4. Updates the scaled variance parameter $\log_{10}(g_m)$.

Step 5. Updates the measurement error rate c. If the other measurement error rate b were also to be estimated as well, an additional step similar to this one would be added.

For instance, to update the dissociating probability of DNMT1 whose current value is ρ_{1d}, we generate a proposal ρ^*_M from a normal distribution with ρ_{1d} as the mean, and standard deviation σ_M. We then compute log likelihoods with the proposal and with the current value, $\log L(\rho^*_M;\cdot)$ and $\log L(\rho_{1d};\cdot)$, respectively. The dot $\cdot$ represents data and current values of other parameters. The proposal is accepted with probability $\min(1, A)$, where the Hastings ratio $A = L(\rho^*_M;\cdot)/L(\rho_{1d};\cdot)$.

The MCMC program, if run long enough, produces the distribution of the parameter of interest. To check whether this was indeed the case, we ran the program from multiple, vastly different starting points. Posterior distributions of λ from different runs were close to each other in all the cases we examined, suggesting that the program worked.

Each run of the MCMC program consisted of 4.2×10^5 iterations, roughly 27-34 CPU hours depending on detailed settings. Samples from every 1600-th iteration were recorded, after the initial 20% of the run (the "burn-in") was discarded. Runs from different starting points are pooled to produce the posterior distributions of the parameters of interest.

4 Extended Viterbi algorithm to infer the top two most likely explanations

The two most likely explanations (with ties) are displayed for some patterns in Figs. 2 and 3 of the main text. The classic Viterbi algorithm [7, 8] finds the most likely combination (explanation) of hidden states to explain the observed pattern under the hidden Markov model. To find the top n most likely explanations, we extend the Viterbi algorithm, adopting the partition scheme introduced in [9] and [10]. The extended Viterbi algorithm is as follows:

For each double-stranded pattern of S CpG sites, do the following:

Step 1. Apply the Viterbi algorithm to find the most likely explanation $H^1 = \{h_1, h_2, \ldots, h_S\}$, where h_s, $s = 1, ..., S$, represents a certain combination of methyltransferase activities and their outcomes at the s-th CpG site. Each h_s represents one of 64 possibilities. A possibility could include, for example, DNMT1 being unassociated with the s-th site, the DNMT3s being associated with the s-th site on the daughter strand but not on the parent strand, the parent-strand CpG being unmethylated before replication and remaining unmethylated after replication, and the daughter-strand CpG becoming methylated. In summary, this possibility represents a de novo methylation event produced by the DNMT3s on the daughter strand at this site.

Step 2. Partition the search space, now excluding H^1, into S subsets, each subset A_s containing

explanations whose first s elements are identical to those of H^1.

$$A_0 = \{h_1^*, h_2^*, \ldots, h_S^*\} \quad (5)$$
$$A_1 = \{h_1, h_2^*, \ldots, h_S^*\} \quad (6)$$
$$A_2 = \{h_1, h_2, h_3^*, \ldots, h_S^*\} \quad (7)$$
$$\vdots$$
$$A_S = \{h_1, h_2, \ldots, h_{S-1}, h_S^*\} \quad (8)$$

where h_s^* are elements different from the s-th element in explanation H^1.

Step 3. Apply the Viterbi algorithm to each subset A_s and compute the probability of the most likely explanation found for this subset. This step generates a list of length S of candidate explanations.

Step 4. Compare the probabilities of the candidate explanations found in Step 3. Explanations with the highest probability are the second most likely explanations (allowing for ties) H^2.

Step 5. Repeat Steps 2-4 to find the n-th most likely explanations.

5 Model checking

As mentioned in the main text, we analyzed the *FMR1* data under a different statistical model in [5], which we tern the Independent Events Model (IEM), Under the IEM, we were interested in CpG site-specific maintenance and de novo methylation probabilities, assuming methylation events to be independent across CpG sites. Our HMM presented in this paper, however, allows methylation events to be dependent across sites. Both models exploit the fact that the data are for each individual molecule, and infer which strand in each double-stranded methylation pattern is the parent or daughter strand, as well as which methylation event may have given rise to hemimethylated CpG dyads, the most informative type of dyads in the data. In addition, we can estimate the probabilities of maintenance and de novo methylation events under both models. Below, we compare the inference for these quantities under the two models, using the *FMR1* data, and demonstrate that these inferences are largely robust to differences in the two models.

5.1 Strand-assignment probabilities

We estimate the probability of assigning the top strand to be the parent strand and the bottom the daughter strand (orientation-specific strand assignment) for each pattern under the two models and display them in Figure 14 here. These probabilities are consistent under both the IEM and the HMM. In addition, the orientation-specific strand-assignment probabilities for the four most informative patterns are all nearly 0 under the HMM, lower than that under the IEM (Supplementary Figure 14). Figures 2 and 3 in the main text report the orientation-nonspecific strand-assignment probabilities, which are max $(p, 1-p)$, where p is the orientation-specific strand-assignment probability and are therefore nearly 1 for each of the four most informative patterns. The more extreme estimated probabilities under the HMM indicates more certainty under this model in assigning the bottom strand in these patterns to be the parent strand. Reduction in uncertainty under the HMM reflects the additional information the HMM gains from accounting for the dependence across sites.

5.2 Inference for hemimethylated CpG dyads

Hemimethylated dyads carry most of the uncertainty in the data and are also most informative about the parameters of interest. Each hemimethylated dyad could have arisen from a failure of maintenance event, a de novo event on the parent strand or on the daughter strand. It could also have been a fully- or unmethylated dyad, but observed as a hemimethylated dyad due to a measurement error. We infer the probability of each possibility for every hemimethylated dyad in the *FMR1* data, using posterior estimates under each model. These probabilities are also mostly consistent under the two models (Supplementary Figure 15).

The main differences in these estimated probabilities under the two models are lower estimated probabilities of parent-strand de novo events and higher estimated probabilities of measurement errors under the HMM than under the IEM (Supplementary Figure 15). We fixed the measurement error rate in the HMM to be the same as that estimated from the IEM and re-ran the HMM. Estimated probabilities of parent-strand de novo events from this run were close to those under the IEM, suggesting that the estimation of the measurement-error rate and that of the parent-strand de novo rate (or the average association/activity level under the HMM) are inversely correlated. This phenomenon is caused by the heterogeneity in the *FMR1* data across CpG sites [5] and the different approaches the two models take to dealing with the heterogeneity. Specifically, we identified previously under the IEM four outlier CpG sites that may have a much higher parent-strand de novo rate than other sites [5]. The HMM, however, effectively assumes a constant parent- or daughter-strand de novo rate across sites. The excess of hemimethylated dyads at the outlier CpG sites cannot be fully explained by a single association/activity rate under the HMM. The parsimonious solution favored by our HMM is then to raise the estimate of other parameters, such as the measurement error rate, to account for the "extra" hemimethylated dyads.

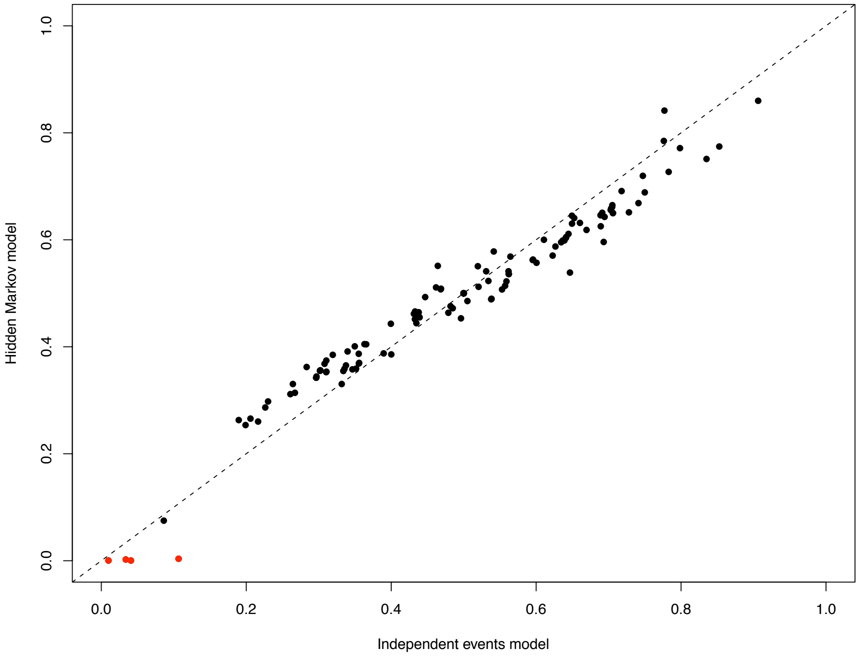

Supplementary Figure 14. Posterior orientation-specific strand-assignment probabilities under the IEM [5] and under the HMM for the *FMR1* data. Each inferred value gives the probability that the top strand was the parent strand and the bottom strand was the daughter strand. Each dot corresponds to one double-stranded methylation pattern. The four red dots correspond to the four most informative patterns.

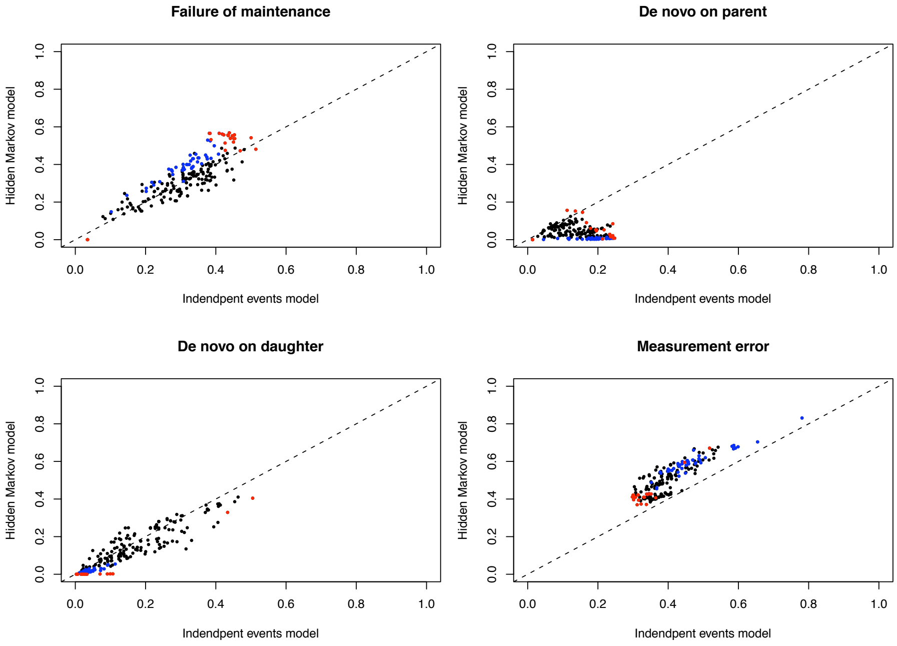

Supplementary Figure 15. Posterior probabilities for the events that gave rise to the hemimethylated CpG dyads in the *FMR1* data, evaluated under the IEM [5] and the HMM. Each dot corresponds to a hemimethylated dyad. Blue dots represent hemimethylated dyads at four CpG sites (sites 10, 14-16), which are identified by the independent-events model to have possibly large de novo rates on the parent strand. Red dots represent hemimethylated dyads on the four most informative patterns (patterns 60, 82, 159 and 165; Figure 2 of the main text). Measurement error in the bottom right panel is due to bisulfite conversion and includes two possibilities: the true state could be a fully methylated site or an unmethylated site. Most measurement errors in the *FMR1* data are of the former type.

5.3 Probabilities of maintenance and de novo methylation events

To compare the results of our HMM with our previous analysis in [5], we compute under the HMM (i) the maintenance methylation probability as $\pi_{1d}\mu_{1d} + \pi_{3d} - \pi_{1d}\mu_{1d}\pi_{3d}$, accounting for contributions from both DNMT1 and the DNMT3s; (ii) the daughter-strand de novo methylation probability as $\pi_{1d}\delta_{1d} + \pi_{3d} - \pi_{1d}\delta_{1d}\pi_{3d}$, also accounting for contributions from both DNMT1 and the DNMT3s; and (iii) the parent-strand de novo methylation probability is identical to π_{3p}, due only to the DNMT3s associated with the parent strand. The resulting estimates are similar to those from the previous analysis (Supplementary Table 3), providing some reassurance that inference of these rates is largely robust to modeling assumptions. The discrepancy in the estimated parent-strand de novo event probabilities is due to a slightly elevated estimate of the measurement error rate c under the HMM compared to [5].

Supplementary Table 3. Estimated mean probabilities of methylation events under the HMM and the IEM for the *FMR1* data.

Mean probability of methylation event	The HMM	The IEM [5]
Maintenance	0.974 (0.962-0.986)	0.976 (0.969-0.983)
De novo on parent strand	0.02 (0.00-0.05)	0.08 (0.04-0.13)
De novo on daughter strand	0.07 (0.03-0.10)	0.07 (0.04-0.11)

Methylation events considered in this table do not distinguish which methyltransferase was at work. Numbers are posterior medians with 80% credible intervals given in parentheses.

References

1. Goyal R, Reinhardt R, Jeltsch A (2006) Accuracy of DNA methylation pattern perservation by the Dnmt1 methyltransferase. Nucleic Acids Res 34: 1182–1188.

2. Vilkaitis G, Suetake I, Klimašauskas S, Tajima S (2005) Processive methylation of hemimethylated CpG sites by mouse Dnmt1 DNA methyltransferase. J Biol Chem 280: 64-72.

3. Rabiner L, Juang B (1986) An introduction to hidden Markov models. ASSP Magazine, IEEE 3: 4–16.

4. Jeffreys H (1946) An invariant form for the prior probability in estimation problems. Proceedings of the Royal Society of London Series A, Mathematical and Physical Sciences 186: 453-461.

5. Fu AQ, Genereux DG, Stöger R, Laird CD, Stephens M (2010) Statistical inference of transmission fidelity of DNA methylation patterns over somatic cell divisions in mammals. Ann Appl Stat 4: 871-892.

6. Liu JS (2001) Monte Carlo Strategies in Scientific Computing. Springer, first edition.

7. Viterbi AJ (1967) Error bounds for convolutional codes and an asymptotically optimum decoding algorithm. IEEE Transactions on Information Theory 13: 260-269.

8. Forney GD (1973) The Viterbi algorithm. Proceedings of the IEEE : 268-278.

9. Nilsson D (1998) An efficient algorithm for finding the m most probable configurations in probabilistic expert systems. Statistics and Computing 8: 159-173.

10. Nilsson D, Goldberger J (2001) Sequentially finding the n-best list in hidden Markov models. Proceedgins of IJCAI : 1280-1285.
